# Strategies to reduce microvascular obstruction and infarct size in patients with ST-elevation myocardial infarction

**DOI:** 10.3389/fcvm.2026.1981540

**Published:** 2026-10-19

**Authors:** Carlotta Rossignoli, Leonardo Portolan, Gabriele Pesarini, Simone Fezzi, Verdiana Galli, Andrea Pacchioni, Domenico Tavella, Flavio Ribichini, Roberto Scarsini, Martina Magistri

**Affiliations:** 1 Department of Medicine, Division of Cardiology, University of Verona, Verona, Italy; 2 Department of Clinical and Molecular Medicine, Sapienza University, Rome, Italy

**Keywords:** cardioprotection, coronary microcirculation, infarct size, ischemia–reperfusion injury, microvascular obstruction, primary percutaneous coronary intervention, ST- elevation myocardial infarction, targeted therapy

## Abstract

Microvascular obstruction (MVO) remains a major challenge in patients with ST-segment elevation myocardial infarction (STEMI), as adequate myocardial reperfusion may not be achieved despite successful epicardial recanalization with primary percutaneous coronary intervention (pPCI). MVO results from multiple interacting mechanisms, including distal embolization, ischemia–reperfusion injury, inflammation, oxidative stress, and microvascular dysfunction, and is associated with larger infarct size and worse clinical outcomes. This review summarizes current and emerging strategies for the diagnosis, prevention, and treatment of MVO in STEMI. Advances in cardiac imaging and invasive or angiography-derived assessment of the coronary microcirculation may improve early identification and risk stratification. Therapeutic approaches include pharmacological and device-based strategies targeting thrombotic burden and distal embolization, reperfusion injury, inflammation, and oxidative stress. However, despite encouraging results for selected interventions, available evidence remains heterogeneous and no specific therapy has become standard of care for MVO. These findings highlight the need to move beyond a uniform approach to MVO. Integration of early diagnosis with identification of the predominant pathophysiological mechanism may provide a framework for future phenotype- and endotype-guided therapeutic strategies delivered within the appropriate therapeutic window. Whether such an approach can improve myocardial reperfusion and clinical outcomes after STEMI requires evaluation in adequately powered prospective trials.

## 1 Introduction

Primary percutaneous coronary intervention (pPCI) remains the cornerstone of reperfusion therapy for patients presenting with ST-segment elevation myocardial infarction (STEMI), as recommended by the current European Society of Cardiology (ESC) guidelines (1). Despite successful restoration of epicardial coronary blood flow, adequate myocardial reperfusion is not achieved in up to 50% of patients owing to coronary microvascular obstruction (MVO), a major determinant of infarct size. Given that the coronary microcirculation accounts for more than 90% of total coronary vascular resistance, MVO plays a central role in limiting myocardial tissue perfusion despite successful epicardial recanalization. Cardiac magnetic resonance studies have consistently demonstrated that MVO is independently associated with adverse left ventricular remodelling, heart failure hospitalization, and increased long-term mortality (2–4).

MVO has a complex and dynamic pathophysiology that involves multiple mechanisms, including oxidative stress and inflammation, platelet–endothelium interactions, altered hemodynamic forces, and autonomic dysregulation. Importantly, microvascular dysfunction is closely interconnected with the epicardial coronary circulation through direct mechanical and humoral interactions (5).

Despite major advances in pPCI, no standardized therapeutic strategy specifically targeting MVO in STEMI has yet been established. Pharmacological and interventional approaches have been extensively investigated, but MVO remains a major unmet clinical need. Targeted prevention and treatment of MVO may therefore represent the next frontier in STEMI management, complementing epicardial reperfusion with pPCI.

In this review, we provide an overview of currently available and emerging pharmacological and device-based therapies targeting the different underlying etiopathogenetic mechanisms of MVO, supporting a mechanism-centred management approach based on accurate diagnosis. Supplementary Table S1 reports a summary of the studies involving the main players of MVO in STEMI, divided by etiopathogenesis. Figure 1 is a summary representation of the therapeutic target for MVO treatment.

**Figure 1:**
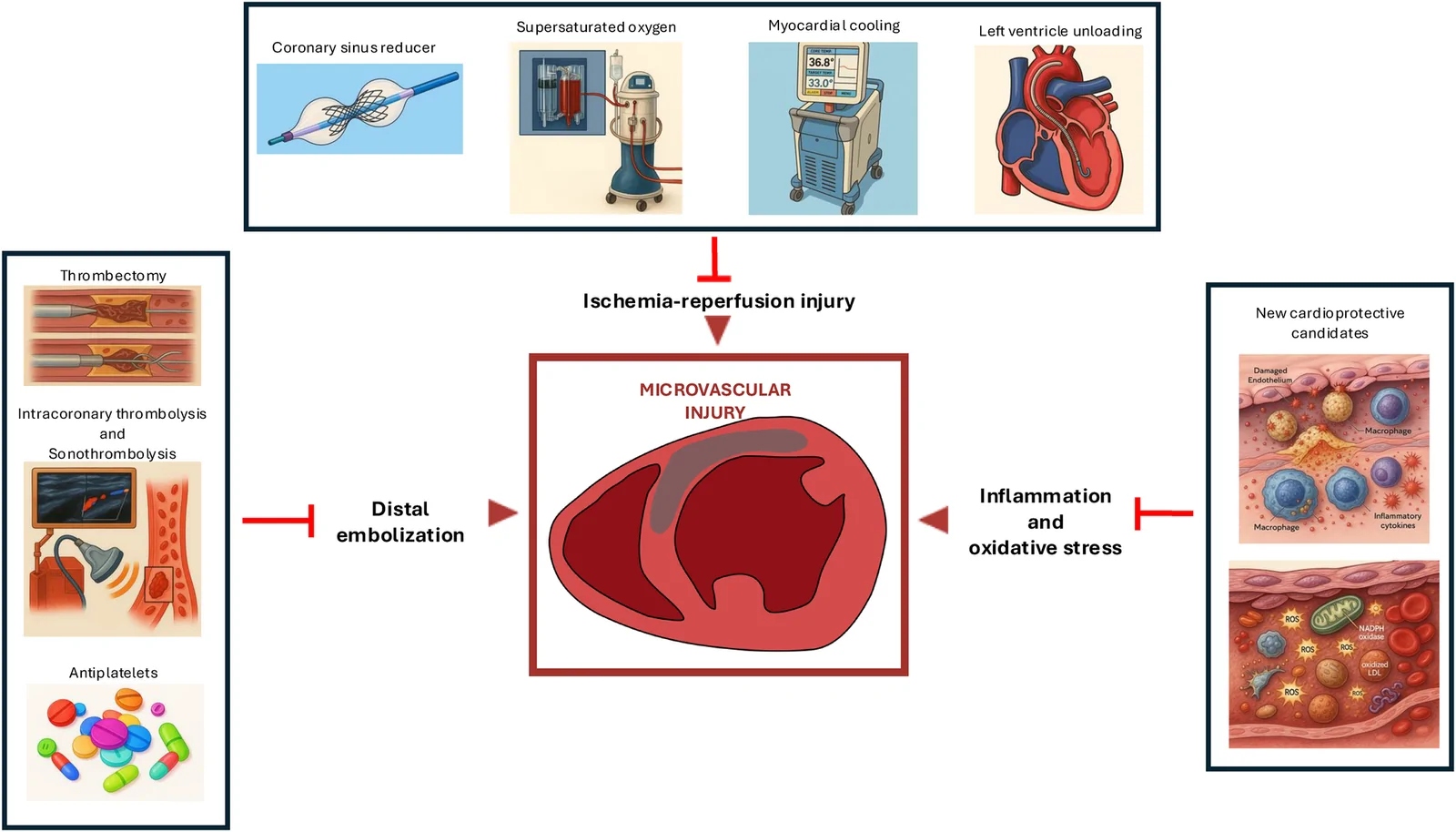
Overview of the pathophysiological mechanisms underlying microvascular obstruction (MVO) in ST-elevation myocardial infarction (STEMI) and current and emerging therapeutic strategies. Distal embolization is targeted by thrombectomy, intracoronary thrombolysis and sonothrombolysis, and antiplatelet agents. Ischemia-reperfusion injury is addressed by coronary sinus reducer, supersaturated oxygen, myocardial cooling, and left ventricular unloading. Ongoing studies aim at new cardioprotective candidates involved in the inflammatory, oxidative and stress responses.

## 2 Literature search and review methodology

This manuscript was designed as a narrative review aimed at providing a clinically oriented and updated overview of the diagnosis, pathophysiology, prevention, and treatment of microvascular obstruction in patients with STEMI.

A literature search was conducted using PubMed, including articles published up to August 2026, to identify relevant studies addressing MVO and its diagnostic and therapeutic approaches. The principal search terms included “microvascular obstruction,” “MVO,” “no-reflow,” “coronary microvascular dysfunction,” “ST-segment elevation myocardial infarction,” “STEMI,” “primary percutaneous coronary intervention,” “myocardial reperfusion,” “ischemia–reperfusion injury,” “distal embolization,” “inflammation,” and “oxidative stress,” together with specific terms related to the diagnostic and therapeutic strategies discussed in this review. Search terms were used alone and in relevant combinations.

Clinical trials, observational studies, systematic reviews and meta-analyses, as well as relevant mechanistic, preclinical, and early feasibility studies of emerging technologies, were considered according to their relevance to the scope of the review.

Particular attention was given to recent evidence and landmark studies relevant to the development of each diagnostic or therapeutic approach.

Given the narrative nature of the review and the substantial heterogeneity of the available evidence in terms of study design, patient populations, interventions, timing, and endpoints, no formal quantitative evidence synthesis or risk-of-bias assessment was performed.

Conflicting or limited evidence is explicitly acknowledged throughout the manuscript, and therapeutic strategies are discussed according to the predominant pathophysiological mechanism of MVO.

Throughout the review, effects on surrogate endpoints, including MVO, infarct size, and indices of microvascular function, are distinguished from effects on clinical outcomes. Improvements in surrogate endpoints are not considered evidence of clinical benefit unless supported by adequately powered studies reporting clinical events.

## 3 Diagnosis of microvascular obstruction

For clarity, the terms MVO, no-reflow, coronary microvascular dysfunction (CMD), and impaired myocardial reperfusion are used here to describe related but distinct phenomena. MVO refers to structural obstruction and injury of the coronary microcirculation despite restoration of epicardial coronary flow and is most specifically characterized by cardiac magnetic resonance. No-reflow refers primarily to the acute failure of adequate myocardial perfusion despite successful epicardial recanalization and represents a clinical/angiographic manifestation that may reflect underlying microvascular injury. CMD is a broader functional abnormality of the coronary microcirculation, which may be assessed using invasive or angiography-derived physiological indices and is not synonymous with MVO. Impaired myocardial reperfusion is used as a broader descriptive term encompassing incomplete restoration of tissue-level perfusion after epicardial reperfusion. Intramyocardial haemorrhage (IMH) should also be distinguished from MVO. IMH results from severe microvascular disruption with extravasation of erythrocytes into the infarcted myocardium and represents a distinct manifestation of severe reperfusion-related microvascular injury. Although IMH frequently occurs within regions of MVO and the two phenomena are closely associated, they are not interchangeable and should be considered distinct pathological and imaging findings.

Currently available diagnostic tools for MVO primarily include cardiac magnetic resonance (CMR) and the invasive wire-based index of microcirculatory resistance (IMR), both of which are limited by technical and logistical constraints. The angiography-derived index of microcirculatory resistance (angio-IMR) has demonstrated both diagnostic and prognostic accuracy comparable to invasive IMR, as well as a similar predictive value to CMR (6). It is becoming increasingly available, facilitating the assessment of microvascular dysfunction (7). Indeed, Duan et al. demonstrated that integrating angio-IMR with CMR-assessed MVO significantly improves risk stratification in STEMI patients undergoing pPCI. While angio-IMR reflects the functional status of the coronary microcirculation, MVO represents the structural/anatomical extent of microvascular injury; therefore, the two parameters provide complementary rather than redundant information. Overall, the study supports a multimodal approach combining invasive physiological assessment and CMR imaging to better characterize coronary microvascular dysfunction after STEMI and to guide future personalized therapeutic strategies (8).

A promising tool for the identification of patients at high risk of MVO following revascularization has been developed by De Maria et al. The age-thrombus burden-index of microcirculatory resistance (ATI) score, based on pre-pPCI IMR, estimates the risk of post-procedural MVO and can be easily and safely assessed prior to stenting. The ATI score has been further validated against CMR (9), predicting the likelihood of MVO and infarct size both acutely and at six-month follow-up. The value of this score lies in its ability to stratify STEMI patients at high risk for MVO early in the catheterization laboratory, predict clinical outcome, and inform decision-making regarding novel additional or alternative therapeutic strategies. However, further studies are required to corroborate its application in clinical practice.

A recent study directly compared photon-counting computed tomography (PCCT) and cardiac magnetic resonance (CMR) for the assessment of myocardial injury in patients with STEMI. Infarct size and MVO quantified by PCCT using late iodine enhancement closely correlated with CMR-derived late gadolinium enhancement, with strong agreement across coronary territories and myocardial viability thresholds. Notably, PCCT accurately identified prognostically relevant MVO and showed excellent segment-level agreement for myocardial viability assessment. Collectively, these findings support the potential role of PCCT as a non-invasive tool to guide diagnosis of MVO and therapeutic decision-making (10).

Preliminary evidence from an early clinical study suggests that the emerging Controlled Flow Infusion (CoFI) system may enable the detection of MVO immediately after pPCI, while also providing the potential to selectively deliver intracoronary therapeutic agents to the vessel affected by MVO. The CoFI system employs the CorFlow software to characterize the relationship between coronary flow and pressure, which is markedly disrupted in the presence of MVO. It consists of a next-generation console, an advanced catheter with a pressure sensor wire, and a single-use infusion cassette. Specifically, the CoFI balloon is positioned within the stented segment, and systolic and diastolic coronary pressures are measured before and after balloon inflation, with a short waiting period to reach the steady-state conditions. Subsequently, a controlled infusion of Ringer's lactate (5, 10, 20, and 30 mL/min) is started, while intracoronary pressure is continuously monitored. At the end of the protocol, the system provides the novel parameter, the CoFI pressure, which is closely related to the presence or absence of MVO. The procedure requires only a few minutes and does not significantly delay STEMI care, as the assessment is performed after stent or balloon deployment. Importantly, it provides an immediate indication of procedural success in treating the culprit lesion. According to the receiver operating characteristic (ROC) analysis, a CoFI pressure threshold of 21.5 mmHg yielded a sensitivity of 95%, a specificity of 91%, and an area under the curve (AUC) of 0.94 for MVO detection (NCT03654573). If confirmed in larger prospective studies, the CorFlow system may represent a new diagnostic tool during pPCI to assess both the presence and the severity of MVO. This could be particularly relevant also from a therapeutic point of view, reinforcing the concept that successful treatment of epicardial coronary arteries alone may be insufficient to restore effective myocardial perfusion.

Figure 2 summarizes the current diagnostic approaches for the assessment of MVO in STEMI, encompassing the conventional invasive and non-invasive modalities as well as the emerging techniques of CoFI. Representative cases further illustrate the relationship between angio-IMR and CMR evidence of MVO.

**Figure 2:**
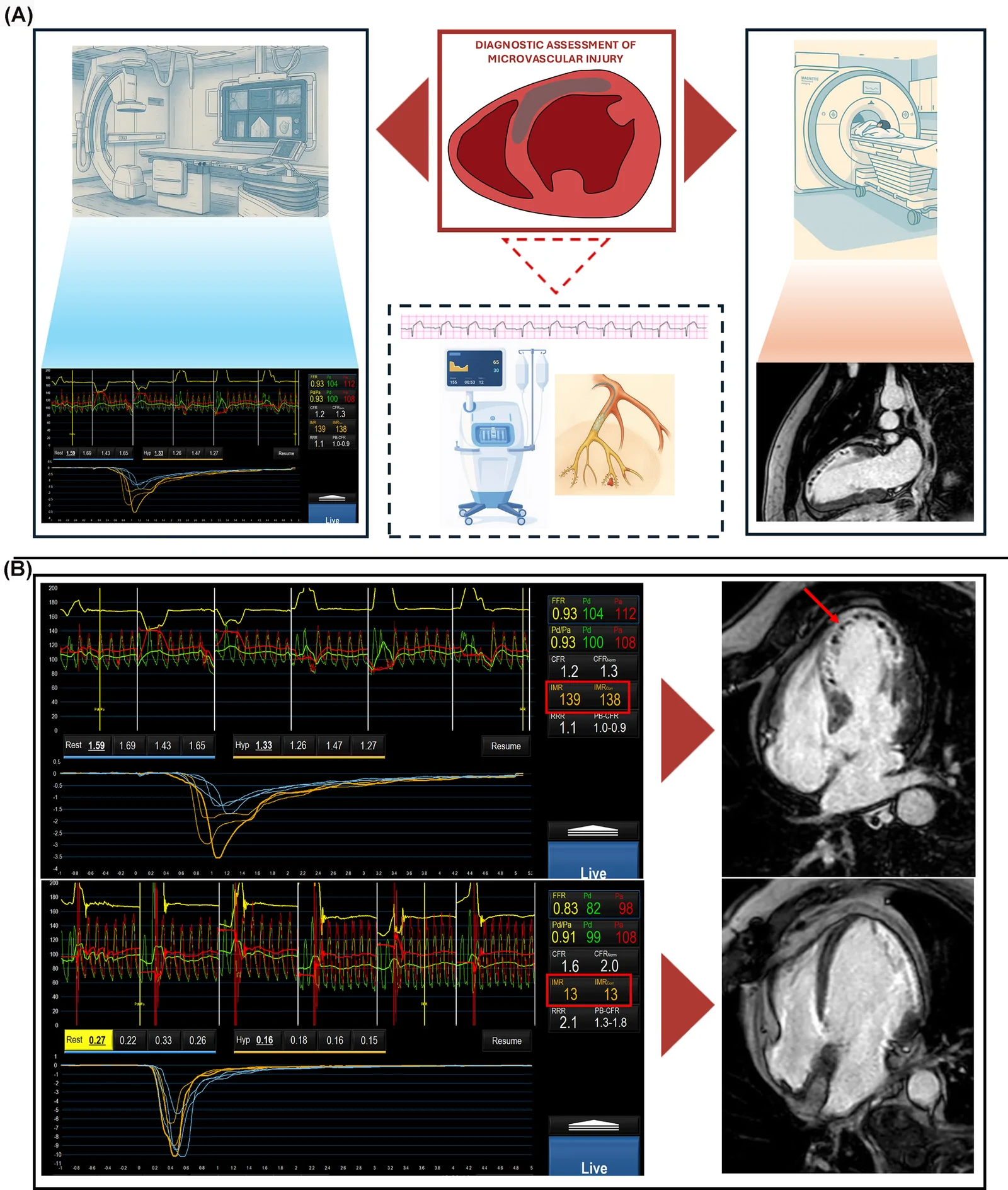
Diagnostic assessment of coronary microvascular obstruction (MVO) in ST-elevation myocardial infarction (STEMI). **(A)** Currently available diagnostic tools for MVO are limited to invasive wire-based coronary physiology (IMR) in the catheterization lab (on the left) and cardiac MRI (on the right). An alternative novel invasive system, the CoFI system (in the middle), has demonstrated the ability to detect MVO immediately after primary PCI and has the potential to selectively deliver therapeutic intracoronary agents selectively in the vessel affected by MVO: if confirmed in future studies, the CorFlow system could represent a new diagnostic tool. **(B)** Representative comparison of a STEMI patient with abnormal angio-IMR and evidence of MVO on CMR versus a STEMI patient with normal angio-IMR and no MVO on CMR.

## 4 Pathophysiological mechanisms of microvascular obstruction and emerging targeted therapies

The principal pathophysiological mechanisms underlying MVO—each selectively addressed by a specific innovative therapeutic strategy—include thrombotic burden and distal embolization, reperfusion injury, inflammation, and oxidative stress. Guideline-directed pharmacological therapies, including antiplatelet agents, statins, and β-blockers in appropriately selected patients, remain fundamental components of STEMI management because of their established cardiovascular benefits. However, these therapies are not specifically approved or recommended for the treatment of MVO, and evidence for a direct MVO-targeted effect remains limited or inconsistent. Indeed, several other pharmacological approaches targeting MVO lack robust clinical evidence in this clinical setting, as illustrated in Supplementary Table S2.

### 4.1 Thrombotic burden and distal embolization

Distal embolization results from the downstream migration of thrombotic and atherosclerotic debris from the culprit plaque, occurring both spontaneously and during pPCI. Embolic material, including platelets, fibrin, plaque components, and cholesterol crystals, obstructs coronary microvessels, leading to microinfarctions. Microembolization triggers cardiomyocyte necrosis and apoptosis and promotes a local inflammatory response which further impairs contractile function in the surrounding viable myocardium (11). Box 1 summarizes the key aspects of distal embolization.

Therapeutic approaches targeting distal embolization are thrombectomy, thrombolytic therapy, and antiplatelet agents.

Box 1 Distal embolization.

- Distal embolization occurs due to downstream migration of thrombotic and atherosclerotic debris.
- Current guidelines discourage routine thrombectomy. It is recommended only as a selective or bailout strategy (high thrombus burden, impaired coronary flow, failure of initial reperfusion, hemodynamic instability).
- Preliminary evidence suggests that sonothrombolysis may improve surrogate measures of myocardial reperfusion when applied before coronary intervention (clinical benefit has not been established and the strategy remains investigational).
- Platelet reactivity in response to dual antiplatelet therapy (DAPT) can predict the extent of myocardial and microvascular damage: effective antiplatelet therapy remains a cornerstone of STEMI management (a specific therapeutic effect on MVO has not been established).

#### 4.1.1 Aspiration and stent-retriever thrombectomy

Selective thrombectomy may improve epicardial flow and reduce distal embolization in high-risk patients, potentially limiting MVO. However, its benefit remains patient- and context-dependent. The most recent ESC and ACC/AHA guidelines recommend thrombectomy only as a bailout or selective strategy, rather than for routine use (1, 12). Specifically, thrombectomy may be considered in the presence of a high thrombus burden, impaired coronary flow, failure of initial reperfusion strategies, hemodynamic instability, and selected anatomical scenarios, such as large proximal vessels or long thrombotic lesions, where stent deployment would increase the risk of distal embolization. Aspiration thrombectomy can be performed manually—which has not demonstrated clinical benefit—or mechanically, using the fully integrated Penumbra System (Penumbra, Inc, Alameda, California). The CHEETAH study (13) evaluated sustained mechanical aspiration thrombectomy before PCI in patients with acute coronary syndrome and a high thrombus burden, supporting its feasibility and showing high rates of thrombus removal, epicardial flow restoration, and normal myocardial perfusion. These findings were further supported by Ismaiel et al. (14), who assessed MVO in patients with reperfused myocardial infarction using CMR, showing that aspiration thrombectomy in addition to PCI may preserve coronary microvascular integrity, reducing the incidence and extent of MVO. Conversely, recent trials have failed to clearly demonstrate its clinical advantage, and the overall utility of aspiration thrombectomy remains controversial. The TASTE trial (15) did not show a reduction in 30-day mortality with routine thrombus aspiration in STEMI patients, and these findings were similarly confirmed by the TOTAL trial (16). In cases of STEMI complicated by massive intracoronary thrombus and refractory no-reflow, stent retriever devices have been proposed as a bail-out mechanical strategy in selected patients, as demonstrated in the RETRIEVE AMI randomized trial (17). Originally developed for acute ischemic stroke, stent retrievers allow mechanical engagement and extraction of organized thrombotic material, potentially limiting distal embolization and MVO. Nevertheless, larger randomized trials are needed before routine adoption. Accordingly, stent retrievers should currently be considered only in highly selected patients and in experienced centres, pending further dedicated studies to define safety, efficacy, and clinical impact.

#### 4.1.2 Intracoronary thrombolysis

Intracoronary thrombolysis is another strategy aimed at reducing the thrombotic burden in STEMI patients, targeting both epicardial and microvascular obstructions with reported improvements in TIMI flow and Myocardial Blush Grade (MBG). However, current evidence regarding adjunctive intracoronary fibrinolytic therapy for the prevention of MVO remains conflicting. Some studies have suggested potential benefits in STEMI patients with high thrombus burden, including improved myocardial perfusion, reduced infarct size, better preservation of left ventricular function, and lower rates of adverse cardiovascular events following administration of low-dose prourokinase or streptokinase during primary PCI. In contrast, larger randomized studies evaluating intracoronary alteplase or prourokinase failed to demonstrate significant improvements in microvascular obstruction or 6-month clinical outcomes compared with standard PCI alone, highlighting the persistent uncertainty surrounding the clinical efficacy of this strategy (18–20).

The combined use of mechanical thrombectomy and intracoronary thrombolysis offers a plausible mechanistic rationale; however, its overall clinical efficacy remains to be established. This strategy—together with potential adjunctive approaches involving novel antiplatelet agents or vasodilators—continues to be investigational.

#### 4.1.3 Sonothrombolysis

Sonothrombolysis is a novel therapeutic strategy based on the delivery of high-mechanical index ultrasound impulses from a diagnostic ultrasound transducer during intravenous microbubble infusion. High mechanical index-induced microbubble cavitation generates shear forces (21) follow that may facilitate the dissolution of both epicardial and microvascular thrombi (22, 23).

The MRUSMI (Microvascular Recovery with Ultrasound in Acute Myocardial Infarction) trial investigated the application of high-mechanical index impulses via a diagnostic ultrasound (DUS) transducer during an infusion of a commercially available microbubble contrast in patients with a first STEMI. When applied before and after PCI, sonothrombolysis was associated with higher epicardial recanalization rates and smaller infarct size, translating into sustained improvements in left ventricular systolic function. Notably, these effects were observed early—before emergent PCI—yet were associated with durable functional benefits and a reduced need for implantable defibrillators during follow-up (24). Consistently, Aguiar et al. (25) reported an association between sonothrombolysis and reduced MVO and improved myocardial dynamics in STEMI patients, with sonothrombolysis emerging as an independent predictor of left ventricular remodelling over time. In contrast, post-PCI sonothrombolysis did not confer additional benefit in the study by El Kadi et al. (26), which reported no significant improvement in ST-segment resolution or infarct size compared to controls.

Overall, preliminary clinical evidence suggests that sonothrombolysis may exert greater effects when applied before coronary intervention; however, the available studies remain relatively small and have primarily evaluated surrogate imaging or functional endpoints. Its effect on clinically meaningful outcomes has not been established, and larger adequately powered randomized trials are required before this strategy can be considered for routine clinical use.

#### 4.1.4 Antiplatelet agents

Platelet inhibition is crucial in the management of STEMI patients and is related to the extent of MVO and thus of myocardial damage. Suboptimal response to antiplatelet therapy has been shown to relate to a greater extent of late gadolinium enhancement as well as MVO assessed with CMR, both associated with a higher rate of major adverse cardiovascular events at 1-year follow-up. These findings suggest an association between effective platelet inhibition and a lower extent of myocardial and microvascular injury. However, whether earlier or more potent platelet inhibition specifically prevents MVO beyond its established role in STEMI management remains uncertain (27).

#### 4.1.5 Intracoronary vasodilators

Intracoronary vasodilators have been extensively investigated for the prevention and treatment of no-reflow during pPCI. Several agents, including adenosine, sodium nitroprusside, calcium-channel blockers, and nicorandil, have been used with the aim of reducing microvascular resistance, relieving microvascular spasm, and improving tissue-level reperfusion. Among these agents, nicorandil combines nitrate-like vasodilatory properties with ATP-sensitive potassium-channel activation, providing a mechanistic rationale for potential protection of the coronary microcirculation during ischemia–reperfusion.

Available evidence suggests that intracoronary vasodilators may improve angiographic and electrocardiographic indices of myocardial reperfusion, although the magnitude and consistency of these effects vary across agents. A network meta-analysis of randomized trials evaluating seven intracoronary agents, including adenosine, diltiazem, nicorandil, nitroprusside, and verapamil, showed that several pharmacological strategies improved surrogate measures of myocardial reperfusion compared with standard primary PCI, although effects on left ventricular function and clinical outcomes were less consistent (28).

Among these agents, nicorandil has shown particularly encouraging effects on myocardial reperfusion. A meta-analysis of 18 randomized controlled trials including 2,055 patients found that nicorandil administered before primary PCI significantly reduced the incidence of no-reflow and improved corrected TIMI frame count, with additional favourable effects on left ventricular ejection fraction during follow-up (29). Consistently, a subsequent meta-analysis of 18 randomized trials reported a lower incidence of no-reflow with nicorandil, together with a reduction in composite major adverse cardiac events (30). However, these findings should be interpreted cautiously because of heterogeneity in nicorandil administration, study populations, definitions of no-reflow, follow-up duration, and composition of clinical endpoints. Moreover, evidence specifically linking nicorandil administration to a reduction in CMR-defined MVO remains limited. Thus, the available evidence supporting nicorandil predominantly relates to angiographic no-reflow and other surrogate measures of myocardial reperfusion rather than to a demonstrated reduction in structural MVO or hard clinical outcomes.

More recently, a 2026 network meta-analysis of 13 randomized trials including 1,674 patients with no-reflow during pPCI compared intracoronary adenosine, epinephrine, nitroprusside, and verapamil. Both verapamil and epinephrine were associated with significantly higher odds of achieving final TIMI grade 3 flow compared with control. ST-segment resolution was also significantly improved with epinephrine, verapamil, and adenosine. Conversely, adenosine and nitroprusside did not show a significant benefit for restoration of final TIMI grade 3 flow. Importantly, none of the evaluated therapies was associated with a significant reduction in major adverse cardiovascular events or all-cause mortality, although these analyses were underpowered for clinical outcomes (31).

Overall, these findings support a potential role for intracoronary vasoactive agents in the management of established angiographic no-reflow during primary PCI, while emphasizing the distinction between restoration of coronary flow, improvement in surrogate measures of myocardial reperfusion, and prevention or treatment of structural MVO. Therefore, improvement in TIMI flow, myocardial blush, or ST-segment resolution should not be considered equivalent to a reduction in CMR-defined MVO or evidence of clinical benefit. Further adequately powered randomized trials incorporating standardized CMR-defined MVO endpoints and clinical outcomes are required before these agents can be considered established MVO-targeted therapies.

#### 4.1.6 Deferred stenting

Deferred stenting has been proposed as an alternative procedural strategy in selected STEMI patients, particularly in the presence of a high thrombus burden. Following restoration of stable coronary flow, postponing stent implantation may allow reduction of thrombus burden and stabilization of the culprit lesion, theoretically limiting distal embolization and microvascular injury associated with immediate stent deployment.

Initial proof-of-concept evidence was encouraging. In the DEFER-STEMI trial, 101 STEMI patients at increased risk of no-reflow were randomized to immediate or deferred stenting 4–16 h after initial reperfusion. Deferred stenting significantly reduced the incidence of no-/slow-reflow and intraprocedural thrombotic events, and was associated with a higher myocardial salvage index at 6 months. However, two patients in the deferred-stenting group experienced recurrent STEMI before the second procedure (32).

Subsequent randomized studies yielded less consistent findings, particularly when microvascular injury was assessed by CMR. In the MIMI trial, deferred stenting did not reduce infarct size and was associated with a numerically greater extent of CMR-defined MVO compared with immediate stenting, with the difference becoming significant after adjustment for the area at risk. Similarly, in the CMR substudy of DANAMI-3-DEFER, involving 510 patients, deferred stenting did not reduce final infarct size, improve myocardial salvage, or reduce the presence of MVO (33, 34).

These findings were further supported by a collaborative meta-analysis of four randomized trials including 1,570 STEMI patients, of whom 797 underwent analysable CMR. Deferred stenting significantly reduced slow/no-reflow compared with immediate stenting, but did not reduce CMR-defined MVO. A non-significant trend toward a higher risk of recurrent ischemia was also observed (35). Notably, an angiographic substudy of DANAMI-3-DEFER subsequently confirmed lower rates of distal embolization and slow/no-reflow with deferred stenting, with larger apparent effects among patients with high thrombus burden, further supporting a potential benefit on procedural rather than structural microvascular endpoints (36).

Importantly, these improvements in angiographic endpoints have not translated into a consistent clinical benefit. In the main DANAMI-3-DEFER trial, which randomized 1,215 patients, routine deferred stenting did not reduce the composite of all-cause mortality, heart failure hospitalization, recurrent myocardial infarction, or unplanned target-vessel revascularization compared with conventional pPCI (37). At 10-year follow-up, deferred stenting similarly did not significantly reduce the composite of all-cause mortality or heart failure hospitalization or all-cause mortality alone, although a lower incidence of heart failure hospitalization was observed and should be considered hypothesis-generating in the context of the neutral primary outcome (38).

Overall, deferred stenting illustrates the distinction between angiographic no-reflow and structural microvascular injury: although delaying stent implantation may reduce distal embolization and slow/no-reflow, particularly in patients with a high thrombus burden, this has not translated into a consistent reduction in CMR-defined MVO, infarct size, or hard clinical outcomes. Therefore, current evidence does not support routine deferred stenting in unselected STEMI patients undergoing primary PCI. Whether a selectively deferred strategy may provide benefit in carefully identified patients with a high thrombotic burden remains to be established in adequately powered randomized trials.

### 4.2 Ischemia-reperfusion injury

Ischemia-reperfusion injury occurs after successful re-opening of an occluded coronary artery when blood flow and oxygen delivery are restored to the ischemic myocardium. Interstitial myocardial oedema occurring after reperfusion leads to small vessel dysfunction, through the compression of capillaries and arterioles, and increases the inflammatory response in the infarct zone. This process contributes to increased morbidity and mortality in STEMI patients and therefore represents a potential therapeutic target. However, the relative contribution of ischemia–reperfusion injury, and consequently the potential benefit of strategies specifically targeting this mechanism, may vary according to ischemic duration and timing of reperfusion. Nevertheless, several strategies aimed at limiting ischemia–reperfusion injury have shown favourable effects on surrogate imaging or physiological endpoints, including infarct size and MVO, in selected studies. However, these findings have been inconsistent across interventions and have not consistently translated into improvements in clinical outcomes. Box 2 summarizes the key aspects of reperfusion injury.

Box 2 Reperfusion injury.

- Ischemia–reperfusion injury occurs after coronary re-opening.
- The use of PiCSO in STEMI patients cannot be recommended in clinical practice.
- More evidence is required for the routine use of the A-FLUX coronary sinus reducer.
- SSO_2_ is a promising device-based technique.
- There are conflicting results in trials for the efficacy of myocardial cooling.
- Mechanical LV unloading remains investigational.
- Beta-blockers are established therapies in selected patients with STEMI, but evidence for a specific protective effect on MVO remains limited and is largely preclinical.

#### 4.2.1 The coronary sinus as a “back door”

Improving blood flow to the deprived myocardium using the coronary sinus (CS) as a “back door” in the absence of antegrade coronary artery blood flow was first described by Pratt in 1,898 with CS retroperfusion of arterial blood in animal models (39). Subsequently, Ido et al. (40) showed that CS occlusion may enhance coronary collateral flow and preserve the ischemic subendocardium. These observations led to the development of three CS-related interventions aimed at increasing perfusion of jeopardized myocardium: CS retroperfusion, CS diameter reduction, and intermittent CS occlusion. Among these, pressure-controlled intermittent Coronary Sinus Occlusion (PiCSO) induces transient pressure elevations through cyclic balloon inflations and deflations, thereby promoting washout of deoxygenated blood from the coronary venous system (41).

The first-in-man PREPARE-PiCSO study and the subsequent PREPARE-RAMSES feasibility study demonstrated that PiCSO could be safely implemented during primary PCI in patients with anterior STEMI and suggested a potential benefit on myocardial recovery in patients receiving adequate therapy, although no significant reduction in infarct size was observed in the overall study population. These encouraging findings prompted the development of prospective randomized trials. However, the multicentre randomized PiCSO-AMI-I trial failed to demonstrate a reduction in infarct size, microvascular obstruction, or intramyocardial haemorrhage compared with conventional primary PCI. The trial was underpowered because recruitment was completed before the planned sample size was reached and PiCSO was associated with longer procedural times and greater contrast use. According to this trial, the use of PiCSO in STEMI patients cannot be recommended in clinical practice, and potential benefit warrants further investigation in adequate studies (42, 43).

#### 4.2.2 The A-FLUX coronary sinus reducer

Importantly, chronic coronary microvascular dysfunction, microvascular angina, and refractory angina represent distinct clinical entities from acute STEMI-associated MVO. Therefore, evidence supporting coronary sinus reduction in chronic ischemic syndromes cannot be directly extrapolated to the prevention or treatment of MVO after STEMI. Nevertheless, the potential effects of coronary sinus narrowing on coronary venous pressure and microvascular resistance provide a mechanistic rationale for investigating this approach in the acute STEMI setting.

The coronary microcirculation, which involves prearterioles, arterioles, and capillaries, is primarily responsible for regulating coronary vascular resistance and myocardial oxygen supply. Accordingly, in patients with CMD, increased microvascular resistance is considered a key determinant of persistent ischemic symptoms. Inducing CS narrowing by the implantation of a CS reducer increases coronary venous pressure, promoting microvascular recruitment. This effect is associated with a reduction in microvascular resistance and an improvement in coronary flow reserve (CFR), ultimately translating into symptomatic relief.

The A-FLUX coronary sinus reducer is a novel self-expandable nitinol device designed to modulate coronary venous outflow. It is fully recapturable up to 50% of deployment and can be repositioned after recapture. It features a small distal tip that facilitates retrieval of the delivery system. The procedure is performed via jugular venous access. After engagement of the CS, CS angiography is performed to identify the optimal implantation site and select the appropriate device size. The delivery system is then advanced to the target location, and the device is deployed by unsheathing. After full deployment, device stability is assessed and confirmed. Following the procedure, DAPT is recommended for six months (44).

The A-FLUX coronary sinus reducer represents a promising investigational approach; however, its safety, feasibility, and potential clinical benefit remain to be established. These aspects are currently being evaluated in the prospective, single-arm, international multicentre early feasibility SERRA-I study (NCT06991322). Accordingly, no conclusions regarding clinical efficacy can currently be drawn.

#### 4.2.3 Supersaturated oxygen (SSO2)

Intracoronary delivery of hyperoxic blood (e.g., supersaturated oxygen, SSO2) has been associated with reduced endothelial oedema, higher capillary vasodilatation, and decreased infarct size in animal models and clinical trials. Indeed, SSO2 provides high levels of dissolved oxygen in plasma, which can readily diffuse into ischemic myocardial tissue while limiting free radical generation, thereby promoting tissue repair and myocardial healing.

Evidence supporting SSO2 therapy in anterior STEMI has progressively evolved over the past two decades. The AMIHOT trial first demonstrated a reduction in infarct size among patients with anterior STEMI treated within 6 h of symptom onset, as assessed by single-photon emission computed tomography after PCI. These findings were subsequently reinforced by the AMIHOT-II trial, which showed that intracoronary SSO2 administration following primary PCI was associated with smaller infarct size without increasing major adverse cardiovascular events. More recently, the IC-HOT study confirmed the safety and provided additional feasibility and safety data of SSO2 therapy delivered through the left main coronary artery after PCI, reporting reductions in infarct size as well as lower rates of adverse clinical outcomes, including death and heart failure hospitalization. Collectively, these findings led to US FDA approval of SSO2 therapy for patients with anterior STEMI involving the left anterior descending artery undergoing primary PCI within 6 h of symptom onset (45–47). These findings suggest a potential beneficial effect of SSO_2_ on coronary microvascular injury; however, a reduction in MVO should not be interpreted as evidence of improved clinical outcomes.

Further evidence was provided by Falah et al., who performed an individual patient-level pooled analysis showing that adjunctive SSO_2_ therapy after successful primary PCI for anterior STEMI was independently associated with a lower extent of microvascular obstruction. More recently, Carlson et al. evaluated SSO_2_ therapy in a small cohort of 19 patients with late-presenting anterior STEMI, reporting feasibility and safety, with lower MVO and fewer adverse clinical events compared with historical controls (48, 49). Given the small sample size, non-randomized design, and use of historical controls, these findings should be considered exploratory and hypothesis-generating. Further adequately powered randomized studies are required to determine whether these observations translate into a clinical benefit in this setting.

#### 4.2.4 Myocardial cooling

Selective intracoronary hypothermia has emerged as a potential cardioprotective strategy aimed at limiting ischemia–reperfusion injury. After crossing the culprit lesion with a pressure–temperature guidewire, an over-the-wire balloon is positioned and inflated proximally to the occlusion to temporarily prevent reperfusion. During this phase, saline at room temperature is infused distal to the balloon, while continuous temperature measurements obtained from the guidewire allow titration of the infusion rate to achieve a distal coronary temperature approximately 6–8 °C below body temperature. Once the target temperature is reached, the balloon is deflated to restore coronary blood flow, and saline infusion is continued during the early reperfusion phase with further adjustments to maintain the desired intracoronary temperature (50).

Although early pilot studies demonstrated the feasibility of therapeutic hypothermia in STEMI, the subsequent COOL AMI-EU Pivotal Trial failed to demonstrate a reduction in infarct size, mainly because systemic cooling significantly prolonged total ischaemic time. Consistently, a recent meta-analysis confirmed that mild hypothermia did not improve infarct size, microvascular obstruction, or clinical outcomes and was associated with clinically relevant treatment delays. In contrast, selective intracoronary hypothermia has emerged as a strategy to minimize reperfusion delay. The EURO-ICE trial demonstrated that this approach is feasible and safe during primary PCI in anterior STEMI; however, it did not significantly reduce infarct size compared with standard care (50–53).

Discrepancies between preclinical studies and randomized clinical trials persist. Uncertainty remains regarding the optimal timing, duration, and depth of cooling, and further clarification is required before hypothermia can be considered a feasible and effective therapeutic strategy.

#### 4.2.5 Mechanical left ventricle unloading

Another promising technique is mechanical left ventricular (LV) unloading, which aims at reducing myocardial oxygen consumption through miniaturized, catheter-mounted axial-flow pumps that can be implanted via femoral access and placed across the aortic valve into the ascending aorta. Preclinical and early clinical evidence suggests that LV unloading may reduce infarct size through decreased myocardial oxygen consumption, improved microcirculatory coronary flow, and attenuation of reperfusion injury (54).

The STEMI-Door-to-Unload (DTU) Pilot Trial was the first study to assess the safety and feasibility of mechanical unloading in STEMI patients, exploring whether a strategy of LV unloading prior to coronary reperfusion may confer clinical benefit. Subsequently, a large-scale, prospective, randomized trial was designed to evaluate whether mechanical unloading of the LV with Impella CP prior to pPCI reduces infarct size and whether this strategy translates into improved clinical outcomes compared with pPCI alone without LV unloading (55). Potential limitations include the invasive nature of the device, challenges in appropriate patient selection, and the limited availability of tools for early and real-time assessment of ventricular loading conditions in the catheterization laboratory.

#### 4.2.6 Beta-blockers

The effects of β-blockers on infarct size and cardiomyocyte protection have already been established by large clinical trials; however, few studies investigated their role in MVO. Data from a large-animal MI model found that intravenous administration of the β1-selective blocker metoprolol before reperfusion reduced MI size (56) and the occurrence of MVO by modulating the inflammatory response during the acute phase of MI and inhibiting the formation of neutrophil-platelet aggregates. Accordingly, metoprolol has been shown to impair neutrophil migration by preventing the morphological changes needed to initiate intercellular interactions and subsequent tissue infiltration (57). Moreover, preclinical studies showed that the third generation β-blockers, such as carvedilol and nebivolol, can protect the coronary microcirculation and thereby reduce infarct size through more potent endothelium-dependent vasodilatory effects. Moreover, intracoronary nebivolol is associated with a significant increase in CFR due to a reduction in resting flow, or due to an increase in maximal coronary flow (58).

Although the effects of β-blockers on infarct size and cardiomyocyte protection have been investigated in large clinical trials, evidence specifically supporting a protective effect on MVO remains limited and is largely derived from preclinical studies.

Current ESC guidelines do not recommend routine intravenous β-blocker administration before primary PCI. However, intravenous β-blockers may be considered in selected haemodynamically stable STEMI patients without signs of acute heart failure or other contraindications (1).

Despite the well-established multiple effects of β-blockers on coronary blood flow—including NO-mediated vasodilation, reduced myocardial oxygen consumption, and improved endothelial function—their impact on the coronary microcirculation remains controversial. Considering the heterogeneity of currently available data, further studies are still needed to confirm their therapeutic effect on MVO.

### 4.3 Inflammation and oxidative stress

Drug-, gene-, and cell-based regenerative therapies are advancing into clinical testing and may become decisive in the management of microvascular injury in acute myocardial infarction, primarily by targeting inflammatory pathways and cellular stress responses.

Inflammation distally into the infarcted myocardium plays an important role in the development of MVO, further contributing to a worse prognosis in affected patients. More specifically, inflammatory mechanisms include the recruitment and activation of inflammatory cells and the formation of neutrophil–platelet aggregates within the coronary microcirculation, leading to microvascular plugging. These processes occur in conjunction with endothelial and capillary injury, characterized by endothelial swelling and the formation of intraluminal endothelial blebs, which further contribute to capillary narrowing and impaired microvascular perfusion. Therefore, targeting inflammation is an important cardioprotective strategy. Box 3 summarizes the key aspects of inflammatory, oxidative and stress responses.

Box 3 Inflammatory, oxidative, stress responses.

- Statins are the cornerstone of IHD drug therapy: they can reduce oxidative stress and inflammation and improve endothelial and microvascular function.
- Anti-inflammatory therapies, including monoclonal antibodies and colchicine, have demonstrated biological or cardiovascular effects in selected clinical settings, but their efficacy as MVO-targeted therapies in acute STEMI remains unproven.
- CRP apheresis remains investigational; preliminary non-randomized evidence suggests potential effects on inflammatory burden, infarct size, and LV function, but clinical benefit has not been established.

#### 4.3.1 Statins

Statins are the cornerstone of therapy for coronary artery disease due to their pleiotropic effects, such as reduction of lipid-rich plaque cores, attenuation of oxidative stress and inflammation, and improvement of endothelial function, with potential benefits for the coronary microcirculation.

At present, the effects of different statin regimens on coronary microcirculatory function in patients with coronary artery disease after PCI have been studied; however, the results are controversial. Some studies have reported that high-intensity statin therapy administered before PCI can significantly improve the coronary microvascular dysfunction after PCI compared with low-dose statin treatment (59). In contrast, other investigations failed to demonstrate a significant benefit of high-intensity statins (60, 61). The conflicting findings may be attributable to considerable heterogeneity in study design, particularly regarding patient selection, clinical setting (elective PCI vs. primary PCI for STEMI), statin regimen and timing of administration, as well as the different methods used to assess coronary microvascular function.

A recent systematic review and meta-analysis demonstrated that, compared with non-high-intensity statin treatment, high-intensity statins significantly improved TIMI flow grade and IMR in patients with coronary artery disease after PCI, suggesting a beneficial effect on coronary microvasculature (62).

Beyond periprocedural indices of coronary microvascular function, Reindl et al. demonstrated that higher admission low-density lipoprotein cholesterol levels were independently associated with a greater extent of microvascular obstruction assessed by cardiac magnetic resonance, as well as with worse clinical outcomes in STEMI patients undergoing primary PCI. These findings further support a potential link between lipid metabolism and microvascular injury following reperfused myocardial infarction. Nevertheless, despite these encouraging findings, the available evidence remains heterogeneous, and adequately powered randomized clinical trials specifically evaluating the impact of intensive lipid-lowering therapy on CMR-defined microvascular obstruction are still lacking (63).

#### 4.3.2 Monoclonal antibodies

Monoclonal antibodies targeting inflammatory pathways represent a biologically plausible investigational approach to limiting microvascular injury after STEMI, primarily by targeting the inflammatory and thrombo-inflammatory pathways involved in ischemia–reperfusion injury. Experimental and early clinical studies have focused on antibodies directed against inflammatory cytokines, adhesion molecules, complement cascade components, and platelet–leukocyte interactions, with the aim of reducing endothelial dysfunction, neutrophil recruitment, microvascular plugging, and capillary damage.

It should be emphasized that current randomized evidence for anti-inflammatory monoclonal antibodies largely derives from studies evaluating cardiovascular outcomes or inflammatory biomarkers rather than MVO-specific endpoints in the acute STEMI setting. Therefore, these data provide a biological and clinical rationale for this therapeutic approach but should not be interpreted as direct evidence of efficacy against MVO.

The CANTOS trial by Ridker et al. (64) showed the effect of Canakinumab, a therapeutic monoclonal antibody targeting interleukin-1b (IL-1b), in lowering the rate of recurrent cardiovascular events in patients with previous myocardial infarction. Treatment with Canakinumab 150 mg reduced the risk of major adverse cardiovascular events (MACE) by 15% [HR: 0.85 (0.74; 0.98)] and participants achieving the highest reduction in IL-6 experienced a 32% reduction in MACE vs. placebo, a 30% reduction in expanded MACE, a 52% reduction in cardiovascular (CV) mortality, a 48% reduction in all-cause mortality with prolonged treatment. In contrast, those with modest decrease in inflammation (IL-6 levels equal to or above 1.65 ng/L) had no reduction in CV risk.

Some years later, the RESCUE trail, a double-blind, randomized trial (65), investigated the effects of Ziltivekimab, a fully human monoclonal antibody against IL-6 ligand, and confirmed the reduction of biomarkers of inflammation and thrombosis relevant to atherosclerosis. The ongoing ARTEMIS trial, an interventional randomized cardiovascular outcome trial (CVOT) has been designed to evaluate the effect of Ziltivekimab vs. placebo, both added to standard of care, on cardiovascular outcomes in patients with acute MI. The aim of the study is to demonstrate safety and efficacy of Ziltivekimab in reducing the risk of adverse cardiovascular events when initiated as early as possible.

However, although preclinical data have shown encouraging effects on infarct size reduction and microvascular preservation, clinical evidence remains limited, and no monoclonal antibody therapy has yet become part of routine clinical practice for MVO management in STEMI patients.

#### 4.3.3 Colchicine

Colchicine has recently gained attention as a potential adjunctive therapy for MVO because of its potent anti-inflammatory properties. By inhibiting microtubule polymerization and suppressing inflammasome activation, colchicine may reduce neutrophil recruitment, platelet–leukocyte interactions, endothelial injury, and the release of pro-inflammatory cytokines involved in ischemia–reperfusion injury.

Indeed, in the COLCOT trial, Tardif et al. demonstrated that colchicine significantly reduced the risk of ischemic cardiovascular events in patients with recent MI within 30 days before enrolment (66). The study showed a 23% reduction in events in the colchicine group in the composite primary endpoint (first event of CV death, resuscitated cardiac arrest, AMI, stroke, or urgent hospitalization for angina requiring coronary revascularization). The Low-Dose colchicine 2 (LoDoCo2) trial investigated the effect and safety of colchicine in patients with stable coronary artery disease and showed a 31% relative reduction in the composite primary endpoint (time to first occurrence CV death, MI, ischemic stroke and ischemia-driven revascularization) (67). Based on data from these randomized placebo-controlled cardiovascular outcome trials, the Food and Drug Administration (FDA) approved colchicine in June 2023 as an anti-inflammatory drug to reduce the risk of MI, stroke, coronary revascularization and CV death in adult patients with established atherosclerotic disease or with multiple risk factors for CV disease.

Importantly, although these randomized trials provide robust evidence for the cardiovascular benefit of colchicine in atherosclerotic disease and after myocardial infarction, they were not designed to assess MVO-specific endpoints. Therefore, the potential role of colchicine in the prevention or treatment of MVO remains mechanistically plausible but has not yet been established by dedicated randomized clinical trials.

#### 4.3.4 C-reactive protein apheresis

C-reactive protein (CRP) apheresis represents a further anti-inflammatory strategy aimed at modulating the inflammatory component of MVO in STEMI. Selective removal of circulating CRP may attenuate ischemia–reperfusion injury by reducing complement activation, endothelial damage, leukocyte recruitment, and microvascular dysfunction. By targeting the amplification of post-infarction inflammation, CRP apheresis could therefore help limit capillary injury and MVO development.

CRP apheresis has emerged as a novel anti-inflammatory strategy to attenuate ischemia–reperfusion injury. In the multicentre, controlled, non-randomized first-in-man CAMI-1 study, Ries et al. demonstrated that CRP apheresis was feasible, safe, and highly effective in rapidly reducing circulating CRP concentrations in patients with STEMI. In patients with large infarcts and pronounced inflammatory responses, CRP apheresis was associated with a dissociation between peak CRP levels and infarct size, together with better preservation of left ventricular function, suggesting a potential cardioprotective effect. Nevertheless, these findings remain hypothesis-generating, and adequately powered randomized clinical trials are required to establish the efficacy of CRP apheresis in improving clinical outcomes (68).

## 5 Evidence-to-practice perspective

From a clinical perspective, MVO-targeted strategies can also be considered according to their timing relative to reperfusion and their predominant therapeutic objective. Supplementary Table 3 summarizes the interventions discussed in this review according to their principal application before, during, or after reperfusion, while distinguishing strategies primarily aimed at preventing MVO from those intended to treat or mitigate established or evolving microvascular injury.

Guideline-directed therapies, including antiplatelet treatment, intensive lipid-lowering therapy, and β-blockers in appropriately selected patients, remain fundamental components of routine STEMI care because of their established cardiovascular benefits; however, their use should not be interpreted as evidence of a specific therapeutic effect on MVO.

Similarly, anti-inflammatory therapies such as colchicine and canakinumab have demonstrated cardiovascular outcome benefits in selected post-MI or atherosclerotic populations, but these trials were not designed to establish MVO-specific efficacy in acute STEMI. Their relevance to MVO therefore remains indirect.

By contrast, most strategies specifically aimed at limiting microvascular injury—including intracoronary thrombolysis, sonothrombolysis, PiCSO, coronary sinus reduction, myocardial cooling, mechanical LV unloading, and CRP apheresis—remain investigational because evidence is predominantly based on surrogate imaging or physiological endpoints, small or non-randomized studies, feasibility investigations, or inconsistent randomized findings. Routine aspiration thrombectomy has not demonstrated clinical benefit and is not recommended, whereas selective mechanical thrombectomy may be considered only in specific bailout settings. SSO_2_ has reached a more advanced stage of clinical development and has demonstrated effects on infarct size and MVO in selected studies; however, robust evidence that these effects translate into improved hard clinical outcomes remains limited.

Intracoronary vasodilators, including nicorandil, may improve angiographic no-reflow and selected surrogate measures of myocardial reperfusion, although evidence for a consistent reduction in CMR-defined MVO or improvement in clinical outcomes remains insufficient. Similarly, deferred stenting may reduce distal embolization and angiographic slow/no-reflow, particularly in patients with a high thrombus burden, but this has not translated into a consistent reduction in CMR-defined MVO or hard clinical outcomes. Accordingly, neither strategy is currently supported for routine use specifically for the prevention or treatment of MVO.

At present, no adjunctive MVO-targeted intervention can be recommended for routine use solely for the prevention or treatment of MVO after STEMI on the basis of demonstrated clinical-outcome benefit. Future adequately powered randomized trials should determine whether mechanism-specific reductions in MVO translate into reductions in heart failure, recurrent cardiovascular events, and mortality.

## 6 Conclusions

MVO plays a cardinal role in STEMI and acute myocardial infarction; however, current diagnostic tools remain limited. Nevertheless, emerging diagnostic techniques may have a relevant impact on prognostic stratification in the near future.

The repeated failure of broadly applied cardioprotective strategies suggests that MVO should no longer be approached as a uniform complication of STEMI, but rather as a heterogeneous, time-dependent syndrome requiring mechanism-based diagnosis and phenotype-guided therapy.

Given its multifactorial pathophysiology, future therapeutic approaches may also require multimodal strategies targeting complementary mechanisms of microvascular injury at different stages of reperfusion, rather than a single universal intervention.

Future trials should therefore move beyond infarct size reduction as a universal endpoint and adopt an endotype-driven design, enrolling patients according to the dominant mechanism of microvascular injury and testing specific or combined therapies within the biologically relevant therapeutic window. As such, MVO remains a subject of ongoing debate and a major challenge, representing an appealing and highly relevant field for future research.

## 7 Future perspectives

Multiple emerging biological targets are currently under investigation to mitigate myocardial injury and microvascular obstruction in STEMI. Haptoglobin phenotyping has been shown to predict MVO and may support risk stratification and personalized therapeutic approaches (69).

Ion channels involved in ischemia-related acidosis and calcium overload, including ASIC1a and selected transient receptor potential (TRP) channels, represent promising cardioprotective targets, although further *in vivo* validation is required. In parallel, cell-based strategies using mesenchymal stem cells and induced pluripotent stem cell–derived pericytes and cardiomyocytes have shown potential to preserve microvascular integrity and promote myocardial repair after infarction.

Overall, these approaches represent promising future directions in the management of MVO but require additional mechanistic insights and robust clinical evidence before translation into routine clinical practice.

## Glossary

ACC: American College of Cardiology
ACS: acute coronary syndrome
AF: atrial fibrillation
AHA: American Heart Association
AMI: acute myocardial infarction
AMPK: AMP-activated protein kinase
angio-IMR: Angiography-derived Index of microcirculatory resistance
ASIC1a: acid-sensing ion channel 1a
ATI: age–thrombus burden–index of microcirculatory resistance
AUC: area under the curve
BID: Twice Daily
CCB(s): calcium channel blocker(s)
CCS: Canadian Cardiovascular Society
CFR: coronary flow reserve
CMR: cardiac magnetic resonance
CMD: coronary microvascular dysfunction
CoFI: controlled flow infusion
CRP: C-reactive protein
CS: coronary sinus
CV: cardiovascular
CVOT: cardiovascular outcome trial
DAPT: dual antiplatelet therapy
DOAC(s): direct oral anticoagulant(s)
DUS: diagnostic ultrasound
ESC: European Society of Cardiology
FDA: Food and Drug Administration
HF: heart failure
HR: hazard ratio
ICD: implantable cardioverter-defibrillator
IL: interleukin
IL-1β: interleukin-1 beta
IL-6: interleukin-6
IMR: index of microcirculatory resistance
IS: infarct size
LV: left ventricle/left ventricular
LVAD: left ventricular assist device
LVEF: left ventricular ejection fraction
MACE: major adverse cardiovascular events
MBF: myocardial blood flow
MBG: myocardial blush grade
MI: myocardial infarction
MIRI: myocardial ischemia–reperfusion injury
MRI: magnetic resonance imaging
MPRI: myocardial perfusion reserve index
MVO: microvascular obstruction
NO: nitric oxide
NOAC(s): non-vitamin K antagonist oral anticoagulant(s)
PCI: percutaneous coronary intervention
PCCT: photon-counting computed tomography
pPCI: primary percutaneous coronary intervention
PiCSO: pressure-controlled intermittent coronary sinus occlusion
RCT: randomized controlled trial
ROC: receiver operating characteristic
SDF-1α: stromal cell-derived factor-1 alpha
SPECT: single-photon emission computed tomography
SSO_2_: supersaturated oxygen
STEMI: ST-segment elevation myocardial infarction
TIA: transient ischemic attack
TIMI: thrombolysis in myocardial infarction
TRP: transient receptor potential.
