# Supplementary material for: Strategies to reduce microvascular obstruction and infarct size in patients with ST-elevation myocardial infarction
Source: Front Cardiovasc Med. 2026 Oct 19;13:1981540. doi: 10.3389/fcvm.2026.1981540 (PMC13725066; doi:10.3389/fcvm.2026.1981540)
Supplement: Supplementary file 1 [file Datasheet2.pdf]

References

1. Byrne RA, Rossello X, Coughlan JJ, Barbato E, Berry C, Chieffo A, et al. 2023 ESC Guidelines for the management of acute coronary syndromes. Eur Heart J. 2023;44(38):3720-826.

2. Betgem RP, de Waard GA, Nijveldt R, Beek AM, Escaned J, van Royen N. Intramyocardial haemorrhage after acute myocardial infarction. Nat Rev Cardiol. 2015;12(3):156-67.

3. de Waha S, Patel MR, Granger CB, Ohman EM, Maehara A, Eitel I, et al. Relationship between microvascular obstruction and adverse events following primary percutaneous coronary intervention for ST-segment elevation myocardial infarction: an individual patient data pooled analysis from seven randomized trials. Eur Heart J. 2017;38(47):3502-10.

4. Scarsini R, Shanmuganathan M, De Maria GL, Borlotti A, Kotronias RA, Burrage MK, et al. Coronary Microvascular Dysfunction Assessed by Pressure Wire and CMR After STEMI Predicts Long-Term Outcomes. JACC Cardiovasc Imaging. 2021;14(10):1948-59.

5. Taqueti VR, Di Carli MF. Coronary Microvascular Disease Pathogenic Mechanisms and Therapeutic Options: JACC State-of-the-Art Review. J Am Coll Cardiol. 2018;72(21):2625-41.

6. Wang L, Travieso A, van der Hoeven N, Lombardi M, van Leeuwen MAH, Janssens G, et al. Angiography-versus wire-based microvascular resistance index to detect coronary microvascular obstruction associated with ST-segment elevation myocardial infarction. Int J Cardiol. 2024;411:132256.

7. Scarsini R, Portolan L, Della Mora F, Marin F, Mainardi A, Ruzzarin A, et al. Angiography-Derived and Sensor-Wire Methods to Assess Coronary Microvascular Dysfunction in Patients With Acute Myocardial Infarction. JACC Cardiovasc Imaging. 2023;16(7):965-81.

8. Duan Y, Yin Q, Yang Y, Miao H, Han S, Chi Q, et al. Integrating angio-IMR and CMR-assessed microvascular obstruction for improved risk stratification of STEMI patients. Sci Rep. 2025;15(1):5470.

9. De Maria GL, Alkhalil M, Wolfrum M, Fahrni G, Borlotti A, Gaughran L, et al. The ATI score (age-thrombus burden-index of microcirculatory resistance) determined during primary percutaneous coronary intervention predicts final infarct size in patients with ST-elevation myocardial infarction: a cardiac magnetic resonance validation study. EuroIntervention. 2017;13(8):935-43.

10. Kotronias RA, Portolan L, Xie C, Ferreira VM, Raman B, Klotz E, et al. Evaluating Acute Ischemic Myocardial Injury With Photon-Counting Computed Tomography. JACC Cardiovasc Imaging. 2026;19(2):284-7.

11. Galli M, Niccoli G, De Maria G, Brugaletta S, Montone RA, Vergallo R, et al. Coronary microvascular obstruction and dysfunction in patients with acute myocardial infarction. Nat Rev Cardiol. 2024;21(5):283-98.

12. Rao SV, O'Donoghue ML, Ruel M, Rab T, Tamis-Holland JE, Alexander JH, et al. 2025 ACC/AHA/ACEP/NAEMSP/SCAI Guideline for the Management of Patients With Acute Coronary Syndromes: A Report of the American College of Cardiology/American Heart Association Joint Committee on Clinical Practice Guidelines. J Am Coll Cardiol. 2025;85(22):2135-237.

13. Mathews SJ, Parikh SA, Wu W, Metzger DC, Chambers JW, Ghali MGH, et al. Sustained Mechanical Aspiration Thrombectomy for High Thrombus Burden Coronary Vessel Occlusion: The Multicenter CHEETAH Study. Circ Cardiovasc Interv. 2023;16(2):e012433.

14. Ismaiel M, Shehata M, Elwasify T, Hosny M, Anwar A, Ahmed TAN, et al. Impact of Aspiration Thrombectomy on Microvascular Obstruction in Patients With ST-segment Elevation Myocardial Infarction. J Saudi Heart Assoc. 2025;37(3):9.

15. Fröbert O, Lagerqvist B, Olivecrona GK, Omerovic E, Gudnason T, Maeng M, et al. Thrombus aspiration during ST-segment elevation myocardial infarction. N Engl J Med. 2013;369(17):1587-97.

16. Jolly SS, Cairns JA, Yusuf S, Meeks B, Pogue J, Rokoss MJ, et al. Randomized trial of primary PCI with or without routine manual thrombectomy. N Engl J Med. 2015;372(15):1389-98.

17. Kotronias RA, Walsh JL, Andreaggi S, Portolan L, Maino A, Marin F, et al. Stent-Retriever Thrombectomy in STEMI With Large Thrombus Burden: The RETRIEVE AMI Randomized Trial. JACC Adv. 2025;4(7):101893.

18. Wang X, Liu H, Wu H, Xiao Y, Bai S, Li X, et al. Safety and efficacy of intracoronary prourokinase administration in patients with high thrombus burden. Coron Artery Dis. 2020;31(6):493-9.

19. Sezer M, Cimen A, Aslanger E, Elitok A, Umman B, Buğra Z, et al. Effect of intracoronary streptokinase administered immediately after primary percutaneous coronary intervention on long-term left ventricular infarct size, volumes, and function. J Am Coll Cardiol. 2009;54(12):1065-71.

20. McCartney PJ, Eteiba H, Maznyczka AM, McEntegart M, Greenwood JP, Muir DF, et al. Effect of Low-Dose Intracoronary Alteplase During Primary Percutaneous Coronary Intervention on Microvascular Obstruction in Patients With Acute Myocardial Infarction: A Randomized Clinical Trial. JAMA. 2019;321(1):56-68.

21. Xie F, Lof J, Everbach C, He A, Bennett RM, Matsunaga T, et al. Treatment of acute intravascular thrombi with diagnostic ultrasound and intravenous microbubbles. JACC Cardiovasc Imaging. 2009;2(4):511-8.

22. Xie F, Lof J, Matsunaga T, Zutshi R, Porter TR. Diagnostic ultrasound combined with glycoprotein IIb/IIIa-targeted microbubbles improves microvascular recovery after acute coronary thrombotic occlusions. Circulation. 2009;119(10):1378-85.

23. Mathias W, Tsutsui JM, Tavares BG, Xie F, Aguiar MO, Garcia DR, et al. Diagnostic Ultrasound Impulses Improve Microvascular Flow in Patients With STEMI Receiving Intravenous Microbubbles. J Am Coll Cardiol. 2016;67(21):2506-15.

24. Aguiar MOD, Tavares BG, Tsutsui JM, Fava AM, Borges BC, Oliveira MT, et al. Sonothrombolysis Improves Myocardial Dynamics and Microvascular Obstruction Preventing Left Ventricular Remodeling in Patients With ST Elevation Myocardial Infarction. Circ Cardiovasc Imaging. 2020;13(4):e009536.

25. El Kadi S, van de Veerdonk MC, Spoormans EM, Verouden NJW, Li S, Xie F, et al. Sonothrombolysis in Patients With ST-Elevation Myocardial Infarction With Electrocardiographic No-Reflow After Percutaneous Coronary Intervention: A Randomized Controlled Trial. J Am Soc Echocardiogr. 2024;37(10):981-92.

26. Massalha E, Oren D, Goitein O, Brodov Y, Fardman A, Younis A, et al. Post-ST-Segment-Elevation Myocardial Infarction Platelet Reactivity Is Associated With the Extent of Microvascular Obstruction and Infarct Size as Determined by Cardiac Magnetic Resonance Imaging. J Am Heart Assoc. 2022;11(3):e020973.

27. Ido A, Hasebe N, Matsuhashi H, Kikuchi K. Coronary sinus occlusion enhances coronary collateral flow and reduces subendocardial ischemia. Am J Physiol Heart Circ Physiol. 2001;280(3):H1361-7.

28. Gibson CM, Ajmi I, von Koenig CL, Turco MA, Stone GW. Pressure-Controlled Intermittent Coronary Sinus Occlusion: A Novel Approach to Improve Microvascular Flow and Reduce Infarct Size in STEMI. Cardiovasc Revasc Med. 2022;45:9-14.

29. Van de Hoef TP, Nolte F, Delewi R, Henriques JP, Spaan JA, Tijssen JG, et al. Intracoronary hemodynamic effects of pressure-controlled intermittent coronary sinus occlusion (PICSO): results from the First-In-Man Prepare PICSO Study. J Interv Cardiol. 2012;25(6):549-56.

30. De Maria GL, Greenwood JP, Zaman AG, Carrié D, Coste P, Valgimigli M, et al. Pressure-Controlled Intermittent Coronary Sinus Occlusion (PiCSO) in Acute Myocardial Infarction: The PiCSO-AMI-I Trial. Circ Cardiovasc Interv. 2024;17(4):e013675.

31. Farjat-Pasos JI, Puri R, Altisent OA, Giannini F, Albert G, Del Portillo J, et al. A-Flux: a novel self-expanding coronary sinus reducer for refractory angina. EuroIntervention. 2024;20(15):e948-e50.

32. O'Neill WW, Martin JL, Dixon SR, Bartorelli AL, Trabattoni D, Oemrawsingh PV, et al. Acute Myocardial Infarction with Hyperoxemic Therapy (AMIHOT): a prospective, randomized trial of intracoronary hyperoxemic reperfusion after percutaneous coronary intervention. J Am Coll Cardiol. 2007;50(5):397-405.

33. Stone GW, Martin JL, de Boer MJ, Margheri M, Bramucci E, Blankenship JC, et al. Effect of supersaturated oxygen delivery on infarct size after percutaneous coronary intervention in acute myocardial infarction. Circ Cardiovasc Interv. 2009;2(5):366-75.

34. David SW, Khan ZA, Patel NC, Metzger DC, Wood FO, Wasserman HS, et al. Evaluation of intracoronary hyperoxemic oxygen therapy in acute anterior myocardial infarction: The IC-HOT study. Catheter Cardiovasc Interv. 2019;93(5):882-90.

35. Falah B, Kotinkaduwa LN, Schonning MJ, Redfors B, de Waha S, Granger CB, et al. Microvascular Obstruction in Patients With Anterior STEMI Treated With Supersaturated Oxygen. J Soc Cardiovasc Angiogr Interv. 2024;3(5):101356.

36. Carlson PE, Schwager S, Feldewerd K, Schmidt S, Campbell M, Bergstedt S, et al. The use of supersaturated oxygen therapy in patients with late-presentation anterior ST-segment elevation myocardial infarction. Int J Cardiol. 2025;436:133429.

37. El Farissi M, Keulards DCJ, van 't Veer M, Zelis JM, Berry C, De Bruyne B, et al. Selective intracoronary hypothermia in patients with ST-elevation myocardial infarction. Rationale and design of the EURO-ICE trial. EuroIntervention. 2021;16(17):1444-6.

38. Noc M, Erlinge D, Neskovic AN, Kafedzic S, Merkely B, Zima E, et al. COOL AMI EU pilot trial: a multicentre, prospective, randomised controlled trial to assess cooling as an adjunctive therapy

to percutaneous intervention in patients with acute myocardial infarction. EuroIntervention. 2017;13(5):e531-e9.

39. Noc M, Laanmets P, Neskovic AN, Petrović M, Stanetic B, Aradi D, et al. A multicentre, prospective, randomised controlled trial to assess the safety and effectiveness of cooling as an adjunctive therapy to percutaneous intervention in patients with acute myocardial infarction: the COOL AMI EU Pivotal Trial. EuroIntervention. 2021;17(6):466-73.

40. Laborante R, Paglianiti DA, Galli M, Patti G, D'Amario D. Impact of Mild Hypothermia As Adjunctive Therapy in Patients With ST-Elevation Myocardial Infarction: A Meta-Analysis and Trial Sequential Analysis of Randomized Controlled Trials. Catheter Cardiovasc Interv. 2025;105(3):543-56.

41. Swain L, Reyelt L, Bhave S, Qiao X, Thomas CJ, Zweck E, et al. Transvalvular Ventricular Unloading Before Reperfusion in Acute Myocardial Infarction. J Am Coll Cardiol. 2020;76(6):684-99.

42. Kapur NK, Kim RJ, Moses JW, Stone GW, Udelson JE, Ben-Yehuda O, et al. Primary left ventricular unloading with delayed reperfusion in patients with anterior ST-elevation myocardial infarction: Rationale and design of the STEMI-DTU randomized pivotal trial. Am Heart J. 2022;254:122-32.

43. Ibanez B, Cimmino G, Prat-González S, Vilahur G, Hutter R, García MJ, et al. The cardioprotection granted by metoprolol is restricted to its administration prior to coronary reperfusion. Int J Cardiol. 2011;147(3):428-32.

44. García-Prieto J, Villena-Gutiérrez R, Gómez M, Bernardo E, Pun-García A, García-Lunar I, et al. Neutrophil stunning by metoprolol reduces infarct size. Nat Commun. 2017;8:14780.

45. Togni M, Vigorito F, Windecker S, Abrecht L, Wenaweser P, Cook S, et al. Does the beta-blocker nebivolol increase coronary flow reserve? Cardiovasc Drugs Ther. 2007;21(2):99-108.

46. He GX, Tan W. High-dose atorvastatin pretreatment could diminishes microvascular impairment in patients undergoing elective percutaneous coronary intervention. J Geriatr Cardiol. 2013;10(4):355-60.

47. Hahn JY, Kim HJ, Choi YJ, Jo SH, Lee S, Ahn KJ, et al. Effects of atorvastatin pretreatment on infarct size in patients with ST-segment elevation myocardial infarction undergoing primary percutaneous coronary intervention. Am Heart J. 2011;162(6):1026-33.

48. Ko YG, Won H, Shin DH, Kim JS, Kim BK, Choi D, et al. Efficacy of early intensive rosuvastatin therapy in patients with ST-segment elevation myocardial infarction undergoing primary percutaneous coronary intervention (ROSEMARY Study). Am J Cardiol. 2014;114(1):29-35.

49. Huang B, Han X, Pan Y, Chen D. A systematic review and meta-analysis of the effect of high-intensity statin on coronary microvascular dysfunction. BMC Cardiovasc Disord. 2023;23(1):370.

50. Reindl M, Reinstadler SJ, Feistritzer HJ, Theurl M, Basic D, Eigler C, et al. Relation of Low-Density Lipoprotein Cholesterol With Microvascular Injury and Clinical Outcome in Revascularized ST-Elevation Myocardial Infarction. J Am Heart Assoc. 2017;6(10).

51. Ridker PM, Everett BM, Thuren T, MacFadyen JG, Chang WH, Ballantyne C, et al. Antiinflammatory Therapy with Canakinumab for Atherosclerotic Disease. N Engl J Med. 2017;377(12):1119-31.

52. Ridker PM, Devalaraja M, Baeres FMM, Engelmann MDM, Hovingh GK, Ivkovic M, et al. IL-6 inhibition with ziltivekimab in patients at high atherosclerotic risk (RESCUE): a double-blind, randomised, placebo-controlled, phase 2 trial. Lancet. 2021;397(10289):2060-9.

53. Tardif JC, Kouz S, Waters DD, Bertrand OF, Diaz R, Maggioni AP, et al. Efficacy and Safety of Low-Dose Colchicine after Myocardial Infarction. N Engl J Med. 2019;381(26):2497-505.

54. Ries W, Torzewski J, Heigl F, Pfluecke C, Kelle S, Darius H, et al. C-Reactive Protein Apheresis as Anti-inflammatory Therapy in Acute Myocardial Infarction: Results of the CAMI-1 Study. Front Cardiovasc Med. 2021;8:591714.

55. Pontone G, Andreini D, Guaricci AI, Guglielmo M, Baggiano A, Muscogiuri G, et al. Association Between Haptoglobin Phenotype and Microvascular Obstruction in Patients With STEMI: A Cardiac Magnetic Resonance Study. JACC Cardiovasc Imaging. 2019;12(6):1007-17.

56. Jolly SS, Niemela K. Aspiration thrombectomy in ST-elevation myocardial infarction. EuroIntervention. 2014;10 Suppl T:T35-8.

57. Srinivasan M, Rihal C, Holmes DR, Prasad A. Adjunctive thrombectomy and distal protection in primary percutaneous coronary intervention: impact on microvascular perfusion and outcomes. Circulation. 2009;119(9):1311-9.

58. Jolly SS, James S, Džavík V, Cairns JA, Mahmoud KD, Zijlstra F, et al. Thrombus Aspiration in ST-Segment-Elevation Myocardial Infarction: An Individual Patient Meta-Analysis: Thrombectomy Trialists Collaboration. Circulation. 2017;135(2):143-52.

59. Thakkar S, Khan S, Nair A, Patel D, Patel H, Desai P, et al. Thrombectomy during Percutaneous Coronary Intervention in Patients With St Elevation Myocardial Infarction. Am J Cardiol. 2025;255:134-42.

60. Mathias W, Tsutsui JM, Tavares BG, Fava AM, Aguiar MOD, Borges BC, et al. Sonothrombolysis in ST-Segment Elevation Myocardial Infarction Treated With Primary Percutaneous Coronary Intervention. J Am Coll Cardiol. 2019;73(22):2832-42.

61. Jeyaprakash P, Pathan F, Sivapathan S, Robledo KP, Madan K, Khor L, et al. Sonothrombolysis Before and After Percutaneous Coronary Intervention Provides the Largest Myocardial Salvage in ST Segment Elevation Myocardial Infarction. J Am Soc Echocardiogr. 2024;37(10):996-1007.

62. Jeyaprakash P, Pathan F, Ozawa K, Robledo KP, Shah KK, Morton RL, et al. Restoring microvascular circulation with diagnostic ultrasound and contrast agent: rationale and design of the REDUCE trial. Am Heart J. 2024;275:163-72.

63. Abo Zeid M, Gadelmawla AF, Khalefa K, Shaban AY. Efficacy of sonothrombolysis as an adjunct to primary percutaneous coronary intervention in ST-segment elevation myocardial infarction: a systematic review and meta-analysis. J Thromb Thrombolysis. 2026;59(2):326-40.

64. El Kadi S, Porter TR, Zanstra M, Siegers A, van Loon RB, Hopman LHGA, et al. Feasibility of sonothrombolysis in the ambulance for ST-elevation myocardial infarction. Int J Cardiovasc Imaging. 2022;38(5):1089-98.

65. Slikkerveer J, Dijkmans PA, Sieswerda GT, Doevendans PA, van Dijk AP, Verheugt FW, et al. Ultrasound enhanced prehospital thrombolysis using microbubbles infusion in patients with acute ST elevation myocardial infarction: rationale and design of the Sonolysis study. Trials. 2008;9:72.

66. Slikkerveer J, Kleijn SA, Appelman Y, Porter TR, Veen G, van Rossum AC, et al. Ultrasound enhanced prehospital thrombolysis using microbubbles infusion in patients with acute ST elevation myocardial infarction: pilot of the Sonolysis study. Ultrasound Med Biol. 2012;38(2):247-52.

67. Lønborg J, Engstrøm T, Ahtarovski KA, Nepper-Christensen L, Helqvist S, Vejlstrup N, et al. Myocardial Damage in Patients With Deferred Stenting After STEMI: A DANAMI-3-DEFER Substudy. J Am Coll Cardiol. 2017;69(23):2794-804.

68. Marquard JM, Engstrøm T, Kelbæk H, Beske RP, Islam U, Høfsten DE, et al. 10-Year Outcomes of Deferred or Conventional Stent Implantation in Patients With STEMI (DANAMI-3-DEFER). Circ Cardiovasc Interv. 2025;18(6):e015369.

69. Carrick D, Oldroyd KG, McEntegart M, Haig C, Petrie MC, Eteiba H, et al. A randomized trial of deferred stenting versus immediate stenting to prevent no- or slow-reflow in acute ST-segment elevation myocardial infarction (DEFER-STEMI). J Am Coll Cardiol. 2014;63(20):2088-98.

70. Kim JS, Lee HJ, Woong Yu C, Kim YM, Hong SJ, Park JH, et al. INNOVATION Study (Impact of Immediate Stent Implantation Versus Deferred Stent Implantation on Infarct Size and Microvascular Perfusion in Patients With ST-Segment-Elevation Myocardial Infarction). Circ Cardiovasc Interv. 2016;9(12).

71. Kook H, Lee HJ, Kim MN, Yu CW, Kim JS, Joo HJ, et al. Effects of deferred versus immediate stenting on left ventricular function in patients with ST elevation myocardial infarction. Medicine (Baltimore). 2021;100(28):e26598.

72. Cassese S, Belle L, Ndrepepa G, Bosson JL, Fusaro M, Lønborg J, et al. Deferred vs Immediate Stenting in Primary Percutaneous Coronary Intervention: A Collaborative Meta-analysis of Randomized Trials With Cardiac Magnetic Resonance Imaging Data. Can J Cardiol. 2018;34(12):1573-80.

73. Mahmoud AN, Saad M, Elgendy AY, Mentias A, Elgendy IY. Deferred or immediate stent implantation for primary percutaneous coronary intervention: A meta-analysis of randomized trials. Catheter Cardiovasc Interv. 2018;91(2):260-4.

74. Qiao J, Pan L, Zhang B, Wang J, Zhao Y, Yang R, et al. Deferred Versus Immediate Stenting in Patients With ST-Segment Elevation Myocardial Infarction: A Systematic Review and Meta-Analysis. J Am Heart Assoc. 2017;6(3).

75. Lee JM, Rhee TM, Chang H, Ahn C, Park TK, Yang JH, et al. Deferred versus conventional stent implantation in patients with acute ST-segment elevation myocardial infarction: An updated meta-analysis of 10 studies. Int J Cardiol. 2017;230:509-17.

76. Bulluck H, Chong JH, Bryant J, Annathurai A, Chai P, Chan M, et al. Effect of Cangrelor on Infarct Size in ST-Segment-Elevation Myocardial Infarction Treated by Primary Percutaneous

Coronary Intervention: A Randomized Controlled Trial (The PITRI Trial). Circulation. 2024;150(2):91-101.

77. van Leeuwen MAH, van der Hoeven NW, Janssens GN, Everaars H, Nap A, Lemkes JS, et al. Evaluation of Microvascular Injury in Revascularized Patients With ST-Segment-Elevation Myocardial Infarction Treated With Ticagrelor Versus Prasugrel. Circulation. 2019;139(5):636-46.

78. Cayla G, Silvain J, O'Connor SA, Collet JP, Montalescot G. An evidence-based review of current anti-platelet options for STEMI patients. Int J Cardiol. 2013;166(2):294-303.

79. Rikken SAOF, Storey RF, Andreotti F, Clemmensen P, Ten Berg JM. Parenteral Antiplatelet Drugs in ST-Elevation Myocardial Infarction: Current Status and Future Directions. Thromb Haemost. 2023;123(2):150-8.

80. Li Z, Li Y, Zhang T, Miao W, Su G. Comparison of the influence of ticagrelor and clopidogrel on inflammatory biomarkers and vascular endothelial function for patients with ST-segment elevation myocardial infarction receiving emergency percutaneous coronary intervention: study protocol for a randomized controlled trial. Trials. 2016;17:75.

81. Li Y, Liang Z, Qin L, Wang M, Wang X, Zhang H, et al. Bivalirudin plus a high-dose infusion versus heparin monotherapy in patients with ST-segment elevation myocardial infarction undergoing primary percutaneous coronary intervention: a randomised trial. Lancet. 2022;400(10366):1847-57.

82. Jenab Y, Sadeghipour P, Mohseni-Badalabadi R, Kaviani R, Hosseini K, Pasebani Y, et al. Direct oral anticoagulants or warfarin in patients with left ventricular thrombus after ST-elevation myocardial infarction: a pilot trial and a prespecified meta-analysis of randomised trials. EuroIntervention. 2025;21(1):82-92.

83. Kido K, Ghaffar YA, Lee JC, Bianco C, Shimizu M, Shiga T, et al. Meta-analysis comparing direct oral anticoagulants versus vitamin K antagonists in patients with left ventricular thrombus. PLoS One. 2021;16(6):e0252549.

84. Albaeni A, Li S, Shan Y, Thakker R, Gaalema DE, Saxena R, et al. Outcomes Associated With Novel Oral Anticoagulants and Warfarin in Patients With Cardiac Thrombus Following ST-Segment Elevation Myocardial Infarction. Am J Cardiol. 2025;236:72-8.

85. Erlinge D, Götberg M, Lang I, Holzer M, Noc M, Clemmensen P, et al. Rapid endovascular catheter core cooling combined with cold saline as an adjunct to percutaneous coronary intervention for the treatment of acute myocardial infarction. The CHILL-MI trial: a randomized controlled study of the use of central venous catheter core cooling combined with cold saline as an adjunct to percutaneous coronary intervention for the treatment of acute myocardial infarction. J Am Coll Cardiol. 2014;63(18):1857-65.

86. Testori C, Beitzke D, Mangold A, Sterz F, Loewe C, Weiser C, et al. Out-of-hospital initiation of hypothermia in ST-segment elevation myocardial infarction: a randomised trial. Heart. 2019;105(7):531-7.

87. El Farissi M, Pijls NHJ, Good R, Engström T, Keeble TR, Beleslin B, et al. A randomised trial of selective intracoronary hypothermia during primary PCI. EuroIntervention. 2024;20(12):e740-e9.

88. Wang YS, Zhang J, Li YF, Chen BR, Khurwolah MR, Tian YF, et al. A pilot clinical study of adjunctive therapy with selective intracoronary hypothermia in patients with ST-segment elevation myocardial infarction. Catheter Cardiovasc Interv. 2018;92(7):E433-E40.

89. Carulli E, McGarvey M, Chabok M, Panoulas V, Rosser G, Akhtar M, et al. Transcoronary cooling and dilution for cardioprotection during revascularisation for ST-segment elevation myocardial infarction: Design and rationale of the STEMI-Cool study. Am Heart J. 2025;282:40-50.

90. Villablanca PA, Rao G, Briceno DF, Lombardo M, Ramakrishna H, Bortnick A, et al. Therapeutic hypothermia in ST elevation myocardial infarction: a systematic review and meta-analysis of randomised control trials. Heart. 2016;102(9):712-9.

91. Alushi B, Ndrepepa G, Lauten A, Lahmann AL, Bongiovanni D, Kufner S, et al. Hypothermia in patients with acute myocardial infarction: a meta-analysis of randomized trials. Clin Res Cardiol. 2021;110(1):84-92.

92. Kapur NK, Alkhouli MA, DeMartini TJ, Faraz H, George ZH, Goodwin MJ, et al. Unloading the Left Ventricle Before Reperfusion in Patients With Anterior ST-Segment-Elevation Myocardial Infarction. Circulation. 2019;139(3):337-46.

93. Esposito ML, Zhang Y, Qiao X, Reyelt L, Paruchuri V, Schnitzler GR, et al. Left Ventricular Unloading Before Reperfusion Promotes Functional Recovery After Acute Myocardial Infarction. J Am Coll Cardiol. 2018;72(5):501-14.

94. Hansen ESS, Madsen TL, Wood G, Granfeldt A, Bøgh N, Tofig BJ, et al. Veno-occlusive unloading of the heart reduces infarct size in experimental ischemia-reperfusion. Sci Rep. 2021;11(1):4483.

95. Azuma R, Shingu Y, Gao J, Wakasa S. Effects of Left Ventricular Unloading on Cardiac Function, Heart Failure Markers, and Autophagy in Rat Hearts with Acute Myocardial Infarction. Int J Mol Sci. 2025;26(9).

96. Drakos SG, Kfoury AG, Hammond EH, Reid BB, Revelo MP, Rasmusson BY, et al. Impact of mechanical unloading on microvasculature and associated central remodeling features of the failing human heart. J Am Coll Cardiol. 2010;56(5):382-91.

97. Thiele H, Møller JE, Henriques JPS, Bogerd M, Seyfarth M, Burkhoff D, et al. Temporary mechanical circulatory support in infarct-related cardiogenic shock: an individual patient data meta-analysis of randomised trials with 6-month follow-up. Lancet. 2024;404(10457):1019-28.

98. Besch L, Schrage B. Unloading in cardiogenic shock: the rationale and current evidence. Curr Opin Crit Care. 2024;30(4):379-84.

99. Zhang P, Wei S, Zhai K, Huang J, Cheng X, Tao Z, et al. Efficacy of left ventricular unloading strategies during venoarterial extracorporeal membrane oxygenation in patients with cardiogenic shock: a protocol for a systematic review and Bayesian network meta-analysis. BMJ Open. 2021;11(10):e047046.

100. De Maria GL, Alkhalil M, Borlotti A, Wolfrum M, Gaughran L, Dall'Armellina E, et al. Index of microcirculatory resistance-guided therapy with pressure-controlled intermittent coronary sinus occlusion improves coronary microvascular function and reduces infarct size in patients with ST-elevation myocardial infarction: the Oxford Acute Myocardial Infarction - Pressure-controlled

Intermittent Coronary Sinus Occlusion study (OxAMI-PICSO study). EuroIntervention. 2018;14(3):e352-e9.

101. Scarsini R, Terentes-Printzios D, Shanmuganathan M, Kotronias RA, Borlotti A, Marin F, et al. Pressure-controlled intermittent coronary sinus occlusion improves the vasodilatory microvascular capacity and reduces myocardial injury in patients with STEMI. Catheter Cardiovasc Interv. 2022;99(2):329-39.

102. van de Hoef TP, Nijveldt R, van der Ent M, Neunteufl T, Meuwissen M, Khattab A, et al. Pressure-controlled intermittent coronary sinus occlusion (PICSO) in acute ST-segment elevation myocardial infarction: results of the Prepare RAMSES safety and feasibility study. EuroIntervention. 2015;11(1):37-44.

103. Mohl W, Komamura K, Kasahara H, Heinze G, Glogar D, Hirayama A, et al. Myocardial protection via the coronary sinus. Circ J. 2008;72(4):526-33.

104. Syeda B, Schukro C, Heinze G, Modaressi K, Glogar D, Maurer G, et al. The salvage potential of coronary sinus interventions: meta-analysis and pathophysiologic consequences. J Thorac Cardiovasc Surg. 2004;127(6):1703-12.

105. Mohl W, Kajgana I, Bergmeister H, Rattay F. Intermittent pressure elevation of the coronary venous system as a method to protect ischemic myocardium. Interact Cardiovasc Thorac Surg. 2005;4(1):66-9.

106. König T, Berliner D, Diekmann J, Akin M, Pfeffer T, Sanchez Martinez C, et al. Impact of intracoronary supersaturated oxygen therapy on microvascular obstruction and infarct size in patients with acute anterior myocardial infarction. EuroIntervention. 2025;21(14):e810-e9.

107. Zeymer U, Hassinger F, Bramlage P, Schafer A, Westermann D, Thiele H, et al. Hyperoxemic oxygen therapy in patients with acute anterior myocardial infarction: HOT-AAMI-design and rationale of a randomized trial. Am Heart J. 2025;286:35-44.

108. Melo PHC, Kachel M, Nowak K, Cheng Y, Wilburn P, Blum D, et al. Effects of Intracoronary SuperSaturated Oxygen Therapy on Myocardial Blood Flow, Microvascular Obstruction, and Myocardial Salvage in ST-Segment-Elevation Myocardial Infarction in Swine Ischemia/Reperfusion Model. J Am Heart Assoc. 2025;14(15):e038891.

109. Kaluza GL, Creech JL, Furer A, Afari ME, Milewski K, Yi GH, et al. Chronic myocardial and coronary arterial effects of intracoronary supersaturated oxygen therapy in swine with normal and ischemic-reperfused myocardium. Sci Rep. 2022;12(1):5785.

110. Verheye S, Jolicœur EM, Behan MW, Pettersson T, Sainsbury P, Hill J, et al. Efficacy of a device to narrow the coronary sinus in refractory angina. N Engl J Med. 2015;372(6):519-27.

111. Foley MJ, Rajkumar CA, Ahmed-Jushuf F, Simader FA, Chotai S, Pathimagaraj RH, et al. Coronary sinus reducer for the treatment of refractory angina (ORBITA-COSMIC): a randomised, placebo-controlled trial. Lancet. 2024;403(10436):1543-53.

112. Tryon D, Corban MT, Alkhouli M, Prasad A, Raphael CE, Rihal CS, et al. Coronary Sinus Reducer Improves Angina, Quality of Life, and Coronary Flow Reserve in Microvascular Dysfunction. JACC Cardiovasc Interv. 2024;17(24):2893-904.

113. Tzanis G, Khokhar AA, Ponticelli F, Gallone G, Palmisano A, Esposito A, et al. Coronary sinus size and ischemia improvement after reducer implantation; "one size to fit them all?". Catheter Cardiovasc Interv. 2021;98(3):E365-E9.

114. Banai S, Ben Muvhar S, Parikh KH, Medina A, Sievert H, Seth A, et al. Coronary sinus reducer stent for the treatment of chronic refractory angina pectoris: a prospective, open-label, multicenter, safety feasibility first-in-man study. J Am Coll Cardiol. 2007;49(17):1783-9.

115. Konigstein M, Shofti R, Schwartz M, Banai S. Coronary sinus reducer for the treatment of chronic refractory angina pectoris-results of the preclinical safety and feasibility study. Catheter Cardiovasc Interv. 2018;92(7):1274-82.

116. Broch K, Anstensrud AK, Woxholt S, Sharma K, Tøllefsen IM, Bendz B, et al. Randomized Trial of Interleukin-6 Receptor Inhibition in Patients With Acute ST-Segment Elevation Myocardial Infarction. J Am Coll Cardiol. 2021;77(15):1845-55.

117. Stähli BE, Klingenberg R, Heg D, Branca M, Manka R, Kapos I, et al. Mammalian Target of Rapamycin Inhibition in Patients With ST-Segment Elevation Myocardial Infarction. J Am Coll Cardiol. 2022;80(19):1802-14.

118. Imbesi A, Greco A, Spagnolo M, Laudani C, Raffo C, Finocchiaro S, et al. Targeting Inflammation After Acute Myocardial Infarction. J Am Coll Cardiol. 2025;86(15):1146-69.

119. Seropian IM, Toldo S, Van Tassell BW, Abbate A. Anti-inflammatory strategies for ventricular remodeling following ST-segment elevation acute myocardial infarction. J Am Coll Cardiol. 2014;63(16):1593-603.

120. Ekeløf S, Jensen SE, Rosenberg J, Gögenur I. Reduced oxidative stress in STEMI patients treated by primary percutaneous coronary intervention and with antioxidant therapy: a systematic review. Cardiovasc Drugs Ther. 2014;28(2):173-81.

121. Villa E, Saso L, Chichiarelli S, Rojas-Solé C, Pinilla-González V, Prieto JC, et al. Antioxidant Cardioprotection in Acute Myocardial Infarction: From Mechanisms to Therapeutic Strategies. Front Biosci (Landmark Ed). 2025;30(8):27678.

122. Xiang M, Lu Y, Xin L, Gao J, Shang C, Jiang Z, et al. Role of Oxidative Stress in Reperfusion following Myocardial Ischemia and Its Treatments. Oxid Med Cell Longev. 2021;2021:6614009.

123. Chen J, Wang B, Meng T, Li C, Liu C, Liu Q, et al. Oxidative Stress and Inflammation in Myocardial Ischemia-Reperfusion Injury: Protective Effects of Plant-Derived Natural Active Compounds. J Appl Toxicol. 2025;45(7):1103-23.

124. Matin E, Ghaffari S, Garjani A, Roshanravan N, Matin S, Mesri Alamdari N, et al. Oxidative stress and its association with ST resolution and clinical outcome measures in patients with ST-segment elevation myocardial infarction (STEMI) undergoing primary percutaneous coronary intervention. BMC Res Notes. 2020;13(1):525.

125. Braunersreuther V, Jaquet V. Reactive oxygen species in myocardial reperfusion injury: from physiopathology to therapeutic approaches. Curr Pharm Biotechnol. 2012;13(1):97-114.

126. Wang W, Kang PM. Oxidative Stress and Antioxidant Treatments in Cardiovascular Diseases. Antioxidants (Basel). 2020;9(12).

127. Liu Y, Wang M, Liang Y, Wang C, Naruse K, Takahashi K. Treatment of Oxidative Stress with Exosomes in Myocardial Ischemia. Int J Mol Sci. 2021;22(4).

128. Amsterdam EA, Wenger NK, Brindis RG, Casey DE, Ganiats TG, Holmes DR, et al. 2014 AHA/ACC Guideline for the Management of Patients with Non-ST-Elevation Acute Coronary Syndromes: a report of the American College of Cardiology/American Heart Association Task Force on Practice Guidelines. J Am Coll Cardiol. 2014;64(24):e139-e228.

129. Bairey Merz CN, Handberg EM, Shufelt CL, Mehta PK, Minissian MB, Wei J, et al. A randomized, placebo-controlled trial of late Na current inhibition (ranolazine) in coronary microvascular dysfunction (CMD): impact on angina and myocardial perfusion reserve. Eur Heart J. 2016;37(19):1504-13.

130. Safdar B, D'Onofrio G, Dziura J, Russell RR, Johnson C, Sinusas AJ. Ranolazine and Microvascular Angina by PET in the Emergency Department: Results From a Pilot Randomized Controlled Trial. Clin Ther. 2017;39(1):55-63.

131. Villano A, Di Franco A, Nerla R, Sestito A, Tarzia P, Lamendola P, et al. Effects of ivabradine and ranolazine in patients with microvascular angina pectoris. Am J Cardiol. 2013;112(1):8-13.

132. Koh JS, Hung OY, Eshtehardi P, Kumar A, Rabah R, Raad M, et al. Microvascular Assessment of Ranolazine in Non-Obstructive Atherosclerosis: The MARINA Randomized, Double-Blinded, Controlled Pilot Trial. Circ Cardiovasc Interv. 2020;13(12):e008204.

133. Golino M, Spera FR, Manfredonia L, De Vita A, Di Franco A, Lamendola P, et al. Microvascular ischemia in patients with successful percutaneous coronary intervention: effects of ranolazine and isosorbide-5-mononitrate. Eur Rev Med Pharmacol Sci. 2018;22(19):6545-50.

134. Schwemer TF, Deutscher N, Diermann N, Böger R, Schwedhelm E, Blankenberg S, et al. Effect of ranolazine on plasma arginine derivatives and urinary isoprostane 8-iso-PGF. Sci Rep. 2019;9(1):5708.

135. Hale SL, Kloner RA. Ranolazine treatment for myocardial infarction? Effects on the development of necrosis, left ventricular function and arrhythmias in experimental models. Cardiovasc Drugs Ther. 2014;28(5):469-75.

136. Qian G, A X, Jiang X, Jiang Z, Li T, Dong W, et al. Early Trimetazidine Therapy in Patients Undergoing Primary Percutaneous Coronary Intervention for ST Segment Elevation Myocardial Infarction Reduces Myocardial Infarction Size. Cardiovasc Drugs Ther. 2023;37(3):497-506.

137. Li Y, Wang D, Hu C, Zhang P, Zhang D, Qin S. Efficacy and Safety of Adjunctive Trimetazidine Therapy for Acute Myocardial Infarction: A Systematic Review and Meta-Analysis. Cardiology. 2016;135(3):188-95.

138. Qu X, Yang P, Jiao L, Yin Y. Trimetazidine: Activating AMPK Signal to Ameliorate Coronary Microcirculation Dysfunction after Myocardial Infarction. Front Biosci (Landmark Ed). 2025;30(1):25565.

139. Farzaei MH, Ramezani-Aliakbari F, Ramezani-Aliakbari M, Zarei M, Komaki A, Shahidi S, et al. Regulatory effects of trimetazidine in cardiac ischemia/reperfusion injury. Naunyn Schmiedebergs Arch Pharmacol. 2023;396(8):1633-46.

140. Ferrari R, Ford I, Fox K, Challeton JP, Correges A, Tendera M, et al. Efficacy and safety of trimetazidine after percutaneous coronary intervention (ATPCI): a randomised, double-blind, placebo-controlled trial. Lancet. 2020;396(10254):830-8.

141. Rosendorff C, Lackland DT, Allison M, Aronow WS, Black HR, Blumenthal RS, et al. Treatment of hypertension in patients with coronary artery disease: a scientific statement from the American Heart Association, American College of Cardiology, and American Society of Hypertension. Circulation. 2015;131(19):e435-70.

142. O'Gara PT, Kushner FG, Ascheim DD, Casey DE, Chung MK, de Lemos JA, et al. 2013 ACCF/AHA guideline for the management of ST-elevation myocardial infarction: a report of the American College of Cardiology Foundation/American Heart Association Task Force on Practice Guidelines. J Am Coll Cardiol. 2013;61(4):e78-e140.

143. Sleight P. Calcium antagonists during and after myocardial infarction. Drugs. 1996;51(2):216-25.

144. Chouairi S, Carrie D, Puel J. Myocardial protection with calcium-channel blockers during ischaemia and reperfusion by PTCA. Eur Heart J. 1995;16 Suppl H:3-8.

145. Ferrari R, Visioli O. Calcium channel blockers and ischaemic heart disease: theoretical expectations and clinical experience. Eur Heart J. 1991;12 Suppl F:18-24.

146. Qian G, Zhang Y, Dong W, Jiang ZC, Li T, Cheng LQ, et al. Effects of Nicorandil Administration on Infarct Size in Patients With ST-Segment-Elevation Myocardial Infarction Undergoing Primary Percutaneous Coronary Intervention: The CHANGE Trial. J Am Heart Assoc. 2022;11(18):e026232.

147. Ahmed TAN, Khidr SS, Abdelrady AA, Demitry SR, El-Naggar HM. Impact of oral nicorandil administration prior to primary percutaneous coronary intervention on no-reflow: a randomized controlled trial. Clin Res Cardiol. 2025.

148. Geng N, Ren L, Xu L, Zou D, Pang W. Clinical outcomes of nicorandil administration in patients with acute ST-segment elevation myocardial infarction undergoing primary percutaneous coronary intervention: a systematic review and meta-analysis of randomized controlled trials. BMC Cardiovasc Disord. 2021;21(1):488.

149. Xu L, Wang L, Li K, Zhang Z, Sun H, Yang X. Nicorandil prior to primary percutaneous coronary intervention improves clinical outcomes in patients with acute myocardial infarction: a meta-analysis of randomized controlled trials. Drug Des Devel Ther. 2019;13:1389-400.

150. Li J, Xu X, Zhou X, Dai J, Ma L, Chen C, et al. Cardiovascular events associated with nicorandil administration prior to primary percutaneous coronary intervention in patients with acute ST-segment elevated myocardial infarction: a systematic review and meta-analysis. Expert Opin Drug Saf. 2019;18(6):537-47.

151. Qi Q, Niu J, Chen T, Yin H, Wang T, Jiang Z. Intracoronary Nicorandil and the Prevention of the No-Reflow Phenomenon During Primary Percutaneous Coronary Intervention in Patients with Acute ST-Segment Elevation Myocardial Infarction. Med Sci Monit. 2018;24:2767-76.

152. Pi SF, Liu YW, Li T, Wang Y, Zhou Q, Liu BJ, et al. Effect of sequential nicorandil on myocardial microcirculation and short-term prognosis in acute myocardial infarction patients undergoing coronary intervention. J Thorac Dis. 2019;11(3):744-52.

153. Zhou J, Xu J, Cheng A, Li P, Chen B, Sun S. Effect of nicorandil treatment adjunctive to percutaneous coronary intervention in patients with acute myocardial infarction: a systematic review and meta-analysis. J Int Med Res. 2020;48(11):300060520967856.

154. Ito N, Nanto S, Doi Y, Kurozumi Y, Natsukawa T, Shibata H, et al. Beneficial effects of intracoronary nicorandil on microvascular dysfunction after primary percutaneous coronary intervention: demonstration of its superiority to nitroglycerin in a cross-over study. Cardiovasc Drugs Ther. 2013;27(4):279-87.

155. Ishii H, Ichimiya S, Kanashiro M, Amano T, Imai K, Murohara T, et al. Impact of a single intravenous administration of nicorandil before reperfusion in patients with ST-segment-elevation myocardial infarction. Circulation. 2005;112(9):1284-8.

156. Huang D, Wu H, Zhou J, Zhong X, Gao W, Ma Y, et al. Intravenous nicorandil during primary percutaneous coronary intervention in patients with ST-Elevation myocardial infarction: Rationale and design of the Clinical Efficacy and Safety of Intravenous Nicorandil (CLEAN) trial. Am Heart J. 2022;244:86-93.

157. Rao SV, O'Donoghue ML, Ruel M, Rab T, Tamis-Holland JE, Alexander JH, et al. 2025 ACC/AHA/ACEP/NAEMSP/SCAI Guideline for the Management of Patients With Acute Coronary Syndromes: A Report of the American College of Cardiology/American Heart Association Joint Committee on Clinical Practice Guidelines. Circulation. 2025;151(13):e771-e862.

158. Ferrari R. Effect of ACE inhibition on myocardial ischaemia. Eur Heart J. 1998;19 Suppl J:J30-5.

159. Shariff N, Dunbar C, Matsumura ME. Relation of pre-event use of inhibitors of the renin-angiotensin system with myocardial infarct size in patients presenting with a first ST-segment elevation myocardial infarction. Am J Cardiol. 2010;106(5):646-9.

160. Pratt FH. The nutrition of the heart through the vessels of Thebesius and the coronary veins. Am J Physiol. 1898;1(1):86–103.

161. Nidorf SM, Fiolet ATL, Mosterd A, Eikelboom JW, Schut A, Opstal TSJ, et al.; LoDoCo2 Trial Investigators. Colchicine in Patients with Chronic Coronary Disease. N Engl J Med. 2020;383(19):1838–1847.

162. Niu X, Zhang J, Bai M, Peng Y, Sun S, Zhang Z. Effect of intracoronary agents on the no-reflow phenomenon during primary percutaneous coronary intervention in patients with ST-elevation myocardial infarction: a network meta-analysis. BMC Cardiovasc Disord. 2018 Jan 10;18(1):3.

163. Oliveri F, Tua L, Raone L, Sparasci FM, Ferlini M, Mandurino-Mirizzi A, De Luca L, Arslan F, Bingen BO, Montero-Cabezas JM. Intracoronary Vasoactive Therapy for No-Reflow During Primary PCI: A Network Meta-Analysis of Randomized Trials. JACC Adv. 2026 Mar;5(3):102599.

164. Belle L, Motreff P, Mangin L, Rangé G, Marcaggi X, Marie A, Ferrier N, Dubreuil O, Zemour G, Souteyrand G, Caussin C, Amabile N, Isaaz K, Dauphin R, Koning R, Robin C, Faurie B, Bonello L, Champin S, Delhaye C, Cuilleret F, Mewton N, Genty C, Viallon M, Bosson JL, Croisille P; MIMI Investigators*. Comparison of Immediate With Delayed Stenting Using the Minimalist Immediate Mechanical Intervention Approach in Acute ST-Segment-Elevation Myocardial Infarction: The MIMI Study. Circ Cardiovasc Interv. 2016 Mar;9(3):e003388.

165. Nepper-Christensen L, Kelbæk H, Ahtarovski KA, Høfsten DE, Holmvang L, Pedersen F, Tilsted HH, Aarøe J, Jensen SE, Raungaard B, Terkelsen CJ, Køber L, Engstrøm T, Lønborg J. Angiographic outcome in patients treated with deferred stenting after ST-segment elevation myocardial infarction-results from DANAMI-3-DEFER. Eur Heart J Acute Cardiovasc Care. 2022 Nov 2;11(10):742-748.

166. Kelbæk H, Høfsten DE, Køber L, Helqvist S, Kløvgaard L, Holmvang L, Jørgensen E, Pedersen F, Saunamäki K, De Backer O, Bang LE, Kofoed KF, Lønborg J, Ahtarovski K, Vejlstrup N, Bøtker HE, Terkelsen CJ, Christiansen EH, Ravkilde J, Tilsted HH, Villadsen AB, Aarøe J, Jensen SE, Raungaard B, Jensen LO, Clemmensen P, Grande P, Madsen JK, Torp-Pedersen C, Engstrøm T. Deferred versus conventional stent implantation in patients with ST-segment elevation myocardial infarction (DANAMI 3-DEFER): an open-label, randomised controlled trial. Lancet. 2016 May 28;387(10034):2199-206.
