# Supplementary material for: Strategies to reduce microvascular obstruction and infarct size in patients with ST-elevation myocardial infarction
Source: Front Cardiovasc Med. 2026 Oct 19;13:1981540. doi: 10.3389/fcvm.2026.1981540 (PMC13725066; doi:10.3389/fcvm.2026.1981540)
Supplement: Supplementary file 2 [file Table1.pdf]

**Target**	**Trial name/identifier**	**Study design and evidence category**	**Population/sample size**	**Primary endpoint**	**Results**			
					MVO	*Infarct size*	*LV function*	*Clinical outcomes and other results*
**Distal embolization**								
Thrombectomy	TAPAS (56-58)	RCT, single-center Randomized clinical evidence	STEMI, n = 1071	Myocardial blush grade	Improved microvascular perfusion	Not directly assessed	Not directly assessed	Lower mortality reported at follow-up, but not confirmed by subsequent larger RCTs; improved microvascular perfusion and lower mortality
	TASTE (56, 58)	RCT, multicenter Randomized clinical evidence	STEMI, n = 7244	All-cause mortality (30d)	No improvement in microvascular perfusion	Not directly assessed	Not directly assessed	No significant reduction in mortality, recurrent MI, stent thrombosis, or revascularization; trend toward reduced rehospitalization for MI/stent thrombosis

	TOTAL (56, 58)	RCT, multicenter Randomized clinical evidence	STEMI, n = 10732	Composite CV death, recurrent MI, shock, HF (180d)	No significant difference in microvascular perfusion	Not directly assessed	Not directly assessed	No significant difference in clinical outcomes; increased stroke risk
	Thakkar et al (2025) (59)	Retrospective cohort Non-randomized clinical evidence	STEMI, n = 50104	30-day and 1-year mortality	Not directly assessed	Not directly assessed	Not directly assessed	No difference in mortality or stroke; higher HF readmission and transfusion rates with thrombectomy
	Jolly et al (2017) (58)	Individual patient meta-analysis Systematic review/meta-analytic evidence	STEMI, n = 18306	CV death (30d), stroke/TIA (30d)	Not directly assessed	Not directly assessed	Not directly assessed	No improvement in CV death overall; reduced CV death in high thrombus burden; increased stroke/TIA risk
	Srinivasan et al (2009) (57)	Review of RCTs Systematic review/meta-analytic evidence	STEMI, n = varied	Surrogate and clinical endpoints	No definitive evidence for efficacy in microvascular injury	Not established	Not established	Conflicting results; no definitive clinical benefit

Sonothrombolysis	Mathias et al. (NCT02410330) (60)	RCT, single-center Randomized clinical evidence	STEMI, first event, n = 100	Infarct size (MRI), LVEF	Not directly quantified as CMR-MVO	Reduced	Improved at 6 months	Not powered for clinical events; improved pre-PCI recanalization, reduced need for ICD
	Jeyaprakash et al. (2024) (61)	RCT, 3-arm, sham-controlled Randomized clinical evidence	STEMI, first event, n = 51	Infarct size (% LV mass, MRI)	Not directly assessed	Trend toward reduction ; not definitive	Not established	No major adverse safety signal, not powered for clinical events; higher myocardial salvage index
	REDUCE (ACTRN12620000807954) (62)	RCT, multicenter, sham-controlled Ongoing randomized clinical evidence	STEMI, planned, n = 150	Infarct size (MRI, day 4±2)	Planned	Ongoing (primary imaging endpoint)	Planned	Ongoing (designed to assess efficacy and cost-effectiveness of sonothromboly sis vs sham)
	Aguiar et al. (24)	RCT, single-center Randomized clinical evidence	STEMI, n = 100	LV remodeling , perfusion defect	Reduced	Reduced early perfusion defect/inf arct-related myocardi al damage	Improved myocardial dynamics and less adverse LV remodeling	Not powered for hard clinical outcomes (sonothrombol ysis was an independent predictor of reduced LV remodeling)

	El Kadi et al. (2024) (25)	RCT, single-center Randomized clinical evidence	STEMI with no-reflow PCI, n = 67	ST-segment resolution	Not directly assessed	No significant effect	No significant effect	No significant difference at 6 months
	Abo Zeid et al. (2025) (63)	Meta-analysis (4 studies) Systematic review/meta-analytic evidence	STEMI, n = 252	LVEF, infarct size, MVO	No significant overall effect	No significant overall effect	Improved (early effect stronger than late effect)	Insufficient evidence for hard clinical benefit (no significant difference in infarct size or microvascular obstruction)
	El Kadi et al. (2022) (64)	Feasibility pilot Pilot/feasibility evidence	STEMI, ambulance, n = 3	Feasibility, safety	Not directly assessed	Not directly assessed	Not directly assessed	Safety/feasibility only; reperfusion before PCI in 1 case
	Sonolysis (NTR161) (65, 66)	Multicenter placebo-controlled RCT protocol with pilot feasibility data Pilot/feasibility and protocol evidence	STEMI, planned, n = 60	TIMI flow, blush grade	Not directly assessed	Not adequately assessed for efficacy	Not directly assessed	Protocol feasible and safe; pilot showed no significant difference in outcomes

Defer stenting	DANAMI-3-DEFER (67, 68)	RCT, multicenter Randomized clinical evidence	STEMI, n =1215	Final infarct size (MRI)	No significant reduction	No significant reduction	No significant difference in follow-up LVEF or LV volumes	No demonstrated clinical benefit attributable to deferred stenting (no reduction in infarct size, MVO, or salvage index overall; benefit in long stent length subgroup)
	DEFER-STEMI (69)	RCT, single-center Randomized clinical evidence	STEMI, high risk, n = 101	No-/slow-reflow (TIMI ≤2)	Not directly CMR-MVO; reduced no-reflow	Not directly assessed	Not established	Not powered for hard clinical outcomes (increased myocardial salvage index)
	INNOVATION (70, 71)	RCT, multicenter Randomized clinical evidence	STEMI, n = 114	Infarct size (MRI)	No overall benefit, reduction reported in anterior-STEMI subgroup	No overall benefit, reduction reported in anterior-STEMI subgroup	Not established	No established hard clinical benefit

	Meta-analysis (Cassese, Mahmoud, Qiao, Lee) (72-75)	Meta-analysis Systematic review/meta-analytic evidence	STEMI, n =1456-2281	Slow/no-reflow, MVO, LVEF, MACE	No consistent reduction	No consistent overall reduction	Possible improvement in long-term LVEF in some analyses	No consistent MACE benefit established
<u>Antiplatelets/anticoagulants</u>	PITRI (NCT03102723) (76)	RCT, double-blind, placebo-controlled Randomized clinical evidence	STEMI, n=209	Infarct size (%LV mass, MRI)	No significant reduction in incidence or extent	No significant reduction	Not a demonstrated treatment effect	Not powered for clinical efficacy (no increase in major bleeding)
	van Leeuwen et al. (NCT02422888) (77)	RCT, open-label Randomized clinical evidence	STEMI, n=110	Index of microcirculatory resistance (IMR)	No demonstrated improvement in microvascular resistance (IMR)	No significant reduction	Not established	Not powered for hard clinical outcomes (no difference in IMR or infarct size; ticagrelor associated with less intramyocardial hemorrhage)
	PLATO (78)	RCT, multicenter Indirect randomized clinical evidence	ACS (STEMI subgroup), n=18,624	CV death, MI, stroke	Not directly assessed (microvascular injury not a primary endpoint)	Not directly assessed	Not directly assessed	Ticagrelor reduced CV events and mortality vs clopidogrel

	TRITON-TIMI 38 (78)	RCT, multicenter Indirect randomized clinical evidence	ACS (STEMI subgroup), n=13,608	CV death, MI, stroke	Not directly assessed (microvascular injury not a primary endpoint)	Not directly assessed	Not directly assessed	Prasugrel reduced CV events and stent thrombosis vs clopidogrel
	INVEST-STEMI (79)	Multicenter cohort Non-randomized clinical evidence	STEMI, n=1,000+	TIMI flow post-PCI	Not directly assessed; microvascular perfusion surrogate improved	Not directly assessed	Not directly assessed	Cangrelor improved myocardial perfusion (TIMI flow) vs tirofiban
	Li et al. (NCT02123004) (80)	RCT, protocol Ongoing randomized clinical evidence	STEMI, n=350 (planned)	Inflammatory biomarkers, endothelial function	Not directly assessed (not direct microvascular injury endpoints)	Not directly assessed	Not directly assessed	Ongoing; designed to assess anti-inflammatory and endothelial effects
	Bivalirudin High-Dose Infusion vs Heparin (81)	RCT, multicenter Indirect randomized clinical evidence	STEMI, n=6,106	All-cause mortality, major bleeding	Not directly assessed (microvascular injury not a primary endpoint, but improved net clinical benefit - clinical mortality/bleeding outcomes only)	Not directly assessed	Not directly assessed	Bivalirudin regimen reduced mortality and major bleeding vs heparin

	T-TIME (20)	RCT, multicenter Randomized clinical evidence	STEMI, n=367	Microvascular obstruction (MRI)	No significant reduction	Not directly assessed	Not directly assessed	No established clinical benefit
	Rivaroxaban vs Warfarin for LV Thrombus (82)	RCT, open-label Indirect randomized clinical evidence	STEMI with LV thrombus, n=50	LV thrombus resolution at 3 months	Not directly assessed	Not directly assessed	Not directly assessed	Numerically higher LV thrombus resolution and less major bleeding with rivaroxaban
	DOACs vs Warfarin for LV Thrombus (83)	Meta-analysis Indirect meta-analytic evidence	LV thrombus post-STEMI, n=227	Stroke/systemic embolism, LV thrombus resolution	Not directly assessed	Not directly assessed	Not directly assessed	No difference in stroke or LV thrombus resolution; lower bleeding with DOACs
	NOACs vs Warfarin for Cardiac Thrombus(84)	Retrospective cohort Indirect non-randomized clinical evidence	STEMI with cardiac thrombus, n=881	Ischemic stroke/TIA, bleeding	Not directly assessed (microvascular injury not directly measured)	Not directly assessed	Not directly assessed	Lower risk of stroke and bleeding with NOACs
**Mitigation of ischemia-reperfusion injury**								

Myocardial cooling	CHILL-MI (NCT01379261) (85)	RCT, multicenter Randomized clinical evidence	STEMI, n=120	Infarct size (%MaR, CMR 4±2d)	Not directly demonstrated	No significant reduction overall; subgroup signal in early anterior STEMI	Not established	HF signal reported, but no definitive clinical benefit
	Testori et al. (NCT01777750) (86)	RCT, open-label, endpoint-blinded Randomized clinical evidence	STEMI, n=101	Myocardial salvage index (CMR)	Not directly demonstrated	Not established	Not established	No significant improvement in salvage index or clinical outcomes; 14 min reperfusion delay
	El Farissi et al. (NCT03447834) (87)	RCT, multicenter Randomized clinical evidence	Anterior STEMI, n=200	Infarct size (%LV mass, CMR 3mo)	Not directly demonstrated	No significant reduction	No significant improvement	Safe and feasible, but no demonstrated clinical efficacy
	Wang et al. (88)	RCT, single-centre, pilot Pilot randomized clinical evidence	STEMI, n=60	IS/MaR (CMR, day 7)	Not directly demonstrated	Reduced IS/MaR in this pilot study (especially in anterior STEMI subgroup)	Not established	No difference in 30d events

	STEMI-Cool (NCT05744415) (89)	RCT, pilot, ongoing Ongoing randomized clinical evidence	STEMI, n=60 (planned)	Feasibility, microvascular resistance, IS (CMR)	Planned assessment of microvascular resistance, not necessarily direct MVO	Planned	Planned	Ongoing; designed to assess microvascular resistance, inflammation, and IS
	Meta-analysis (Villablanca et al.) (90)	Systematic review/meta-analysis Systematic review/meta-analytic evidence	STEMI, 6 RCTs, n=819	MACE, infarct size, HF/PO	Not directly demonstrated	No overall reduction (reduced infarct size in anterior STEMI)	Not established	No MACE or mortality benefit
	Meta-analysis (Alushi et al.) (91)	Meta-analysis, 9 RCTs Systematic review/meta-analytic evidence	Acute MI, n=1012	All-cause death, infarct size	Not directly demonstrated	No overall reduction	Not established	No difference in mortality or IS overall; smaller IS in patients achieving target temperature
	Meta-analysis (Laborante et al.) (40)	Meta-analysis, 10 RCTs Systematic review/meta-analytic evidence	STEMI, n=varied	IS, MVO, LVEF, TIMI 3 flow	No significant effect	No significant effect	No significant effect	No significant all-cause mortality benefit; increased infection, stent thrombosis, AF

LV unloading	DTU-STEMI Pilot (NCT03000270) (92)	RCT, multicenter, exploratory Pilot randomized clinical evidence	Anterior STEMI, n=50	Safety (MACE 30d), infarct size (CMR)	Assessed by CMR; no randomized evidence of MVO reduction established	No significant overall reduction in original pilot comparison	Exploratory follow-up parameters; no definitive treatment benefit	Feasible and safe; no significant difference in MACE
	STEMI-DTU Pivotal (NCT03947619) (42)	RCT, multicenter, ongoing Ongoing randomized clinical evidence	Anterior STEMI, n=668 (planned)	Infarct size (%LV mass, CMR 3-5d)	Planned endpoint	Planned efficacy endpoint	Planned secondary assessment	Designed to assess infarct size, microvascular obstruction, and 1-year CV outcomes (planned clinical follow-up, no completed efficacy evidence)
	Swain et al. (41)	Preclinical, swine model Preclinical evidence	Swine AMI	Infarct size, mitochondrial function	Not reported as direct MVO	Reduced	Not established (mitochondrial/metabolic preservation rather than clinical LV function)	Not assessed (preclinical)

	Esposito et al. (93)	Preclinical, swine model Preclinical evidence	Swine AMI	Infarct size, LV function (28d)	Not directly assessed	Reduced	Improved/preserved LV function and reduced scar	Not assessed (preclinical)
	Hansen et al. (94)	Preclinical, porcine model Preclinical evidence	Porcine ischemia-reperfusion	Infarct size, LV function	Not directly reported	Reduced	Preserved LVEF (and increased myocardial salvage)	Not assessed (preclinical)
	Azuma et al. (95)	Preclinical, rat model Preclinical evidence	Rat AMI	Cardiac function, autophagy markers	Not directly assessed	Not directly assessed	Improved cardiac function/remodeling-related markers	Not assessed (preclinical)
	Drakos et al. (96)	Human, LVAD cohort Indirect non-randomized clinical evidence	End-stage HF, n=15	Microvascular density, fibrosis	Not directly assessed (microvascular density assessed)	Not directly assessed	Human LVAD remodeling evidence, not STEMI-specific	LVAD unloading increased microvascular density, decreased lumen area, increased fibrosis (no applicable acute-STEMI clinical outcome)

	Thiele et al. (97)	Meta-analysis, RCTs Indirect meta-analytic evidence	AMI with cardiogenic shock	6-month mortality, complications	Not directly assessed	Not directly assessed	Not directly assessed	Mortality benefit in selected cardiogenic-shock population at 6 months
	Besch & Schrage (98, 99)	Review/meta-analysis Indirect systematic review/meta-analytic evidence	Cardiogenic shock	Mortality, myocardial recovery	Not directly assessed	Not directly assessed	Not directly assessed (myocardial recovery discussed)	Clinical outcomes in cardiogenic shock; indirect evidence
**Enhancement of coronary MV function**								
PiCSO	PiCSO-AMI-I (NCT03625869) (30)	RCT, multicenter Randomized clinical evidence	Anterior STEMI, n=145	Infarct size (CMR, 5 days)	No significant reduction	No significant reduction	Not established	No device-related safety excess through 6 months; no demonstrated clinical efficacy
	OxAMI-PICSO (100)	IMR-guided cohort Non-randomized clinical evidence	Anterior STEMI, n=105	IMR, infarct size (CMR, 6 months)	Direct MVO effect not established; microvascular function improved via lower IMR	Reduced at 6 months	Not established	Not powered for clinical outcomes (reduced IMR at 24–48h and infarct size at 6 months)

	Scarsini et al. (101)	Matched cohort Non-randomized clinical evidence	STEMI (anterior/inferior), n=36 PICSO, n=72 controls	IMR, RRR, infarct size (CMR, 6 months)	Reduced MVO frequency	Reduced at follow-up	Not established	Not powered for hard clinical outcomes
	Prepare RAMSES (102)	Safety/feasibility cohort Pilot/feasibility evidence	Anterior STEMI, n=30	Infarct size, myocardial function (CMR)	Not established	Exploratory reduction in successfully treated patients	Not established (myocardial function assessed but no robust comparative efficacy established)	Feasibility limited
	Mohl et al. (103)	Long-term follow-up cohort Non-randomized clinical evidence	STEMI, n=34	Reinfarction, MACE (48 months)	Not directly assessed	Not directly assessed	Not directly assessed	Lower reinfarction and MACE reported, but non-randomized/historical-control evidence
	Syeda et al. (104, 105)	Meta-analysis (preclinical) Preclinical meta-analytic evidence	Animal MI models, n=213	Infarct size (% area at risk)	Not established	Reduced in animal models	Not principal outcome	Not assessed (preclinical)

Supersaturated oxygen	AMIHOT II (NCT00175058) (33)	RCT, multicenter Randomized clinical evidence	Anterior STEMI, n=301	Infarct size (SPECT)	Not the principal demonstrated endpoint	Reduced	Not a demonstrated primary effect	Safety MACE at 30 days; no superiority for clinical events established
	König et al. (106)	Prospective cohort Non-randomized clinical evidence	Anterior AMI, n=40	Infarct size (CMR, SPECT), MVO	Reduced	Reduced	Not established (no definitive LV-function benefit established)	Not powered for clinical events
	HOT-AAMI (NCT06742684) (107)	RCT, multicenter, ongoing Withdrawn before enrollment (no clinical evidence)	Anterior STEMI, n=1,266 (planned) No patients enrolled	Planned composite: all-cause mortality or HF events (12–48 months)	No results	No results	No results	Withdrawn before enrollment, no clinical outcome data
	Melo et al. (preclinical) (108)	Animal model, randomized Preclinical evidence	Swine STEMI, n=12	MVO (histology), MBF, salvage index	Reduced	Not principal result	Not established (improved myocardial blood flow/salvage rather than LV function)	Not assessed (preclinical)

	Kaluza et al. (preclinical) (109)	Animal model, randomized Preclinical evidence	Swine MI, n=40	Safety, infarct size (histology)	Not directly assessed	Trend toward reduction; not definitive	Improved contractility reported	Not assessed (preclinical)
A-flux coronary sinus reducer	COSIRA (NCT01205893) (110)	RCT, double-blind, sham-controlled Indirect randomized clinical evidence	Refractory angina, n=104	≥2 CCS class improvement at 6 months	Not directly assessed	Not directly assessed	Not directly assessed	Significant improvement in angina and quality of life; no direct STEMI or acute microvascular injury data
	ORBITA-COSMIC (111)	RCT, placebo-controlled Indirect randomized clinical evidence	Refractory angina, n=50	Myocardial blood flow (CMR), daily angina episodes	Not directly assessed	Not directly assessed	Not an acute-STEMI remodeling study	No significant improvement in stress MBF in ischaemic segments; reduction in angina episodes; not STEMI
	Tryon et al. (112)	Phase II, open-label Indirect non-randomized clinical evidence	ANOCA with microvascular dysfunction, n=30	Change in microvascular function (CFR, CBF) at 120 days	Not directly assessed	Not directly assessed	Not directly assessed	Significant improvement in CFR, CBF, angina class, and quality of life; not STEMI

	Tzanis et al. (113)	Prospective cohort Indirect non-randomized clinical evidence	Refractory angina, n=15	MPRI (CMR) at 4 months	Not directly assessed	Not directly assessed	Not directly assessed	No hard clinical outcome evidence (greater ischemia improvement in patients with smaller CS size; not STEMI)
	Banai et al. (114)	Prospective, open-label Indirect pilot/feasibility evidence	Refractory angina, n=15	Safety, feasibility, angina score	Not directly assessed	Not directly assessed	Not directly assessed	Feasibility/symptom improvement only
	Konigstein et al. (115)	Preclinical, swine model Preclinical evidence	Swine with/without ischemia, n=34	Safety, feasibility, ischemia parameters	Not directly assessed	Not directly applicable	Not established	Not assessed (preclinical)
**New cardioprotective agents**								
<u>Inflammatory response</u>	ASSAIL-MI (NCT03004703) (116)	RCT, double-blind, placebo-controlled Randomized clinical evidence	STEMI, n=199	Myocardial salvage index (MRI, 3–7 days)	Less extensive MVO	No statistically significant reduction in final	Not established (at primary publication)	Adverse events similar; not powered for hard clinical efficacy (Tocilizumab increased

						infarct size		myocardial salvage index)
	CLEVER-ACS (NCT01529554) (117)	RCT, double-blind, placebo-controlled Randomized clinical evidence	STEMI, n=150	Change in MI size (MRI, 12h–5d to 30d)	No significant reduction	No significan t reduction	No significant effect on LV-volume remodelling	No clinical efficacy demonstrated; adverse events similar
	CANTOS (not STEMI-specific) (118)	RCT, double-blind, placebo-controlled Indirect randomized clinical evidence	Post-MI, n=10,061	MACE (composite)	Not directly assessed	Not directly assessed	Not directly assessed	Not STEMI-specific; reduced recurrent events, but no direct microvascular injury data
	COLCOT (not STEMI-specific) (118)	RCT, double-blind, placebo-controlled Indirect randomized clinical evidence	Recent MI, n=4,745	MACE (composite)	Not directly assessed	Not directly assessed	Not directly assessed	Not STEMI-specific; reduced ischemic events, but no direct microvascular injury data

	Anakinra STEMI pilot (118, 119)	RCT, open-label, pilot Pilot randomized clinical evidence	STEMI, n=99	CRP reduction, LV remodeling	Not directly assessed	Not established	No significant benefit	Reduced CRP and adverse remodeling in pooled/secondary analyses; no direct microvascular injury endpoint
	IL-1 blockade (various) (118, 119)	RCTs, pilot Pilot randomized clinical evidence	STEMI, small n	Inflammatory markers, LV function	Not directly assessed	Not established	No significant difference; exploratory remodeling signals across small pilots	Reduced inflammation, improved remodeling; no direct microvascular injury endpoint
<u>Oxidative and stress response</u>	Edaravone STEMI RCT (120)	RCT Randomized clinical evidence	STEMI, n=60	Infarct size, LVEF	Not directly assessed	Reduced	Improved	No demonstrated hard clinical benefit
	Allopurinol STEMI RCT (120, 121)	RCT Randomized clinical evidence	STEMI, n=72	Infarct size, cardiac enzymes	Not directly assessed	Reduced	Cardiac-function improvement reported	No established hard clinical benefit
	Nicorandil STEMI RCT (120, 121)	RCT Randomized clinical evidence	STEMI, n=120	TIMI flow, MVO, LVEF	Reduced/improved microvascular perfusion	Not established	Improved	No established hard clinical benefit
	N-acetylcysteine STEMI RCT (120, 122)	RCT Randomized	STEMI, n=50	Oxidative stress	Not directly assessed	Not established	Not established	No significant clinical

		clinical evidence		markers, LVEF				outcome benefit
	Vitamin C STEMI RCT (120, 122)	RCT Randomized clinical evidence	STEMI, n=40	Oxidative stress markers, LVEF	Not directly assessed	No significant effect	Not established	No established clinical benefit
	Atorvastatin STEMI RCT (120, 123)	RCT Randomized clinical evidence	STEMI, n=80	Infarct size, LVEF	Not directly assessed	Reduced	Improved	No hard clinical benefit established
	Deferoxamine STEMI RCT (120)	RCT Randomized clinical evidence	STEMI, n=30	Oxidative stress markers	Not directly assessed	Not established	Not established	No significant clinical outcome benefit
	Natural Compounds AMI RCT (124)	RCT Randomized clinical evidence	AMI, n=100	MIRI markers, LVEF	Not directly assessed	Not established	Improved cardiac function reported	No demonstrated hard clinical benefit
	Exosome Therapy (preclinical) (125)	Preclinical/Review Preclinical evidence	MI models	Oxidative stress, infarct size	Not established	Reduced in preclinical models	Not established (preclinical functional effects only)	Not assessed (preclinical)
	Oxidative Stress Biomarker Study (126, 127)	Observational Non-randomized clinical evidence	STEMI, n=276	Oxidative stress, LVEF, MACE	Not directly assessed	Not directly assessed	Association with better LVEF	Association with fewer adverse events; observational, not treatment effect
