# Supplementary material for: Strategies to reduce microvascular obstruction and infarct size in patients with ST-elevation myocardial infarction
Source: Front Cardiovasc Med. 2026 Oct 19;13:1981540. doi: 10.3389/fcvm.2026.1981540 (PMC13725066; doi:10.3389/fcvm.2026.1981540)
Supplement: Supplementary file 3 [file Table2.pdf]

Table 2. Pharmacological approaches targeting MVO lack robust clinical evidence in this clinical setting

Drug	Current practice	Clinical trials/ongoing studies
Ranolazine	No randomized controlled trials have specifically addressed ranolazine for microvascular injury in STEMI, and it is not recommended for this indication by the American College of Cardiology or the American Heart Association in their guidelines (128).	Small randomized trials and mechanistic studies in patients with microvascular angina and coronary microvascular dysfunction, have shown mixed results: some improvement in angina frequency and myocardial perfusion reserve in select subgroups, but no consistent benefit in overall microvascular function or clinical outcomes (129-132).<br><br>In patients with recent PCI and persistent ischemia, ranolazine improved time to ischemia during stress testing but did not affect coronary microvascular function or diastolic function (133). Experimental animal models suggest ranolazine may reduce infarct size and oxidative stress, but these findings have not been confirmed in clinical STEMI populations (134, 135).
Trimetazidine	Trimetazidine is not currently recommended by major US or European STEMI guidelines as standard therapy for microvascular obstruction. Available evidence suggests potential beneficial effects on microvascular injury and CMR-derived surrogate endpoints when used early in selected STEMI patients, but the evidence base remains limited and has not established a clinical-outcome benefit (136-140).	In a randomized, double-blind, placebo-controlled trial, trimetazidine given prior to PCI and continued for 12 months was associated with a lower incidence of CMR-defined microvascular obstruction (29.7% vs 52.7%, p=0.005), smaller infarct size, and a higher myocardial salvage index at 7 days compared with placebo; however, no significant difference in heart failure readmissions was observed (136).<br><br>Meta-analyses of adjunctive trimetazidine in acute myocardial infarction have reported reductions in composite MACE, but no significant effect on short- or long-term all-cause mortality, recurrent MI, or in-hospital adverse events; these data should not be

		interpreted as definitive evidence of efficacy specifically for MVO (137).<br><br>Mechanistic studies suggest that trimetazidine may improve coronary microcirculatory and endothelial barrier function after ischemia-reperfusion injury, including through AMPK-related pathways, but these findings remain supportive rather than confirmatory for clinical MVO treatment (138, 139).
<u>Calcium channel blockers</u>	Calcium channel blockers are not recommended as a treatment for microvascular injury in patients with ST-elevation myocardial infarction (STEMI): lack of efficacy and potential harm in the acute STEMI setting, with use limited to specific indications such as relief of ischemia or rate control in atrial fibrillation when beta blockers are contraindicated, and only in the absence of severe LV dysfunction (141, 142).	Experimental models and early-phase clinical studies suggested that CCBs may attenuate ischemia-reperfusion injury and improve angiographic or other surrogate measures of no-reflow by modulating calcium overload, improving collateral flow, and protecting endothelial and mitochondrial function when administered prior to ischemia or during early reperfusion (143-145).<br>However, these effects have not translated into consistent clinical benefit in randomized controlled trials.
<u>Nicorandil</u>	Nicorandil is not established as a specific therapy for microvascular obstruction in STEMI. Randomized trials and meta-analyses suggest potential benefit mainly for angiographic no-reflow and other surrogate measures of myocardial reperfusion, whereas evidence for a consistent reduction in CMR-defined MVO and improvement in hard clinical outcomes remains limited (146-155).	The CHANGE trial, a multicenter, double-blind RCT, reported that intravenous nicorandil before reperfusion reduced infarct size at 5-7 days and 6 months, lowered the incidence of angiographic no-reflow/slow-flow (9.2% vs 26.3%), and improved left ventricular ejection fraction compared with placebo (146). These findings support a potential effect on reperfusion-related surrogate endpoints but do not by themselves establish efficacy for CMR-defined MVO or clinical outcomes.<br><br>A recent RCT of oral nicorandil (20 mg before PCI, then 20 mg BID for 3 months) reported lower

		rates of no-reflow, improved myocardial blush grade and ST-segment resolution, and favorable changes in left ventricular function and a composite MACE endpoint at 3 months (147). The short follow-up and use of predominantly angiographic, electrocardiographic, and composite endpoints warrant cautious interpretation.<br><br>Meta-analyses of RCTs have reported lower rates of angiographic no-reflow (OR 0.34-0.47), improved TIMI flow and LVEF, and reductions in composite cardiovascular events or heart failure after STEMI (148-150, 153). However, heterogeneity in administration protocols, definitions of no-reflow, follow-up, and clinical endpoints limits the certainty and generalizability of these findings. Combination intracoronary and intravenous administration has appeared favorable in some analyses, and intracoronary nicorandil has improved post-PCI IMR compared with nitroglycerin (148, 152, 154). Overall, the evidence predominantly concerns angiographic no-reflow and surrogate measures of microvascular function rather than a demonstrated reduction in CMR-defined MVO or hard clinical outcomes.<br><br>Nicorandil is generally well tolerated, with hypotension among the principal adverse effects (151, 154). The ongoing CLEAN trial may provide further information on longer-term efficacy and safety (156).
<u>Ace-inhibitors</u>	ACE-Is are not specifically recommended as a therapeutic target for microvascular	The evidence supporting angiotensin-converting enzyme inhibitors as a therapeutic target for microvascular obstruction in

	obstruction in patients with ST-elevation myocardial infarction. The benefit of ACE inhibitors is attributed to their effects on ventricular remodelling, afterload reduction, and neurohormonal modulation, rather than a direct effect on microvascular obstruction. The American College of Cardiology does not recommend ACE inhibitors specifically for microvascular obstruction, and there is no guideline-supported dosing for this indication (157).	STEMI is indirect, with benefits primarily mediated through improved endothelial function, reduced infarct size, and prevention of adverse remodeling, rather than a direct effect on microvascular obstruction as measured by contemporary imaging or invasive techniques (158, 159).
