# Supplementary material for: Strategies to reduce microvascular obstruction and infarct size in patients with ST-elevation myocardial infarction
Source: Front Cardiovasc Med. 2026 Oct 19;13:1981540. doi: 10.3389/fcvm.2026.1981540 (PMC13725066; doi:10.3389/fcvm.2026.1981540)
Supplement: Supplementary file 4 [file Table3.pdf]

Timing	Main therapeutic objective	Strategy	Prevention vs treatment of MVO	Clinical interpretation
**BEFORE REPERFUSION**	Reduce thrombotic burden and distal embolization before PCI	Antiplatelet therapy	Prevention	Established component of STEMI care, but a specific MVO-preventive effect has not been established.
	Promote early recanalization and reduce distal embolization	Sonothrombolysis	Prevention	Investigational; evidence is mainly based on surrogate endpoints, without established clinical-outcome benefit.
	Attenuate ischemic injury before restoration of coronary flow	Myocardial cooling	Prevention	Investigational; randomized evidence has not demonstrated consistent benefit.
	Reduce myocardial oxygen demand and ischemia-reperfusion injury	Early beta-blockade	Prevention	Established in selected STEMI patients for guideline-directed indications; specific MVO-preventive benefit remains uncertain.
**DURING REPERFUSION / PCI**	Reduce distal embolization in selected patients with high thrombus burden or procedural no-reflow	Selective/bailout mechanical thrombectomy	Prevention / acute treatment of no-reflow	Routine aspiration thrombectomy is not supported; selective mechanical thrombectomy may be considered as a bailout approach.
	Reduce distal embolization associated with immediate stent implantation	Deferred stenting	Prevention	Routine use is not supported by randomized evidence.

	Reduce myocardial workload before full reperfusion and attenuate reperfusion injury	Mechanical LV unloading	Prevention	Investigational; definitive efficacy remains under evaluation.
	Modulate coronary venous pressure and improve microvascular function	PiCSO	Prevention / acute mitigation	Investigational; available clinical evidence has not established hard-outcome benefit.
	Modify coronary venous pressure and microvascular resistance	Coronary sinus reducer / A-FLUX	Prevention / acute mitigation	Investigational in acute STEMI; rationale is partly extrapolated from chronic microvascular dysfunction/refractory angina.
**AFTER REPERFUSION**	Improve microvascular perfusion and limit evolving microvascular injury	Supersaturated oxygen (SSO2)	Treatment / mitigation of evolving MVO	Relatively advanced clinical development, with effects on surrogate endpoints in selected studies; robust hard-outcome evidence remains limited.
	Attenuate post-reperfusion inflammatory injury	Anti-inflammatory therapies	Treatment / mitigation	MVO-specific evidence remains limited or indirect; some agents have outcome evidence in broader post-MI populations.
	Reduce oxidative stress and reperfusion-related cellular injury	Antioxidant/stress-response therapies	Treatment / mitigation	Predominantly experimental or supported by small clinical studies; no established MVO-specific clinical benefit.
	Reduce circulating inflammatory mediators	CRP apheresis	Treatment / mitigation	Early non-randomized/feasibility evidence; hypothesis-generating.
	Promote vascular and myocardial repair	Regenerative, cell-, gene-, and exosome-based therapies	Treatment / repair	Predominantly preclinical or early-stage evidence; investigational.
